# Selection, Phenotyping and Identification of Acid and Hydrogen Peroxide Producing Bacteria from Vaginal Samples of Canadian and East African Women

**DOI:** 10.1371/journal.pone.0041217

**Published:** 2012-07-23

**Authors:** John J. Schellenberg, Tim J. Dumonceaux, Janet E. Hill, Joshua Kimani, Walter Jaoko, Charles Wachihi, Jane Njeri Mungai, Margo Lane, Keith R. Fowke, T. Blake Ball, Francis A. Plummer

**Affiliations:** 1 Department of Medical Microbiology, University of Manitoba, Winnipeg, Canada; 2 Department of Community Health Sciences, University of Manitoba, Winnipeg, Canada; 3 Department of Pediatrics, University of Manitoba, Winnipeg, Canada; 4 Saskatoon Research Centre, Agriculture & Agri-Food Canada, Saskatoon, Canada; 5 Department of Veterinary Microbiology, University of Saskatchewan, Saskatoon, Canada; 6 Department of Medical Microbiology, University of Nairobi, Nairobi, Kenya; 7 National Microbiology Laboratory, Canadian Science Centre for Human and Animal Health, Winnipeg, Canada; Rush University, United States of America

## Abstract

The common but poorly understood condition known as bacterial vaginosis (BV) increases vulnerability to HIV infection and is associated with the absence of H_2_O_2_-producing *Lactobacillus*. Vaginal lactic acid bacteria (LAB) produce anti-HIV factors such as organic acids and hydrogen peroxide (H_2_O_2_), and may bind and inactivate HIV particles during scavenging of mannose. These factors define potential criteria for initial selection of candidate probiotics to block heterosexual transmission of HIV. Therefore, the primary goal of this study was to characterize acid production on mannose and H_2_O_2_ production in vaginal isolates from Canadian adolescents (192 isolates, 16 individuals) and commercial sex workers in Nairobi, Kenya (576 isolates, 96 individuals). Selection of isolates from H_2_O_2_-detecting media suggested an idiosyncratic individual-level profile and extensive phenotypic diversity, including the identification of a subset of “double-strong” acid- and H_2_O_2_-producers with phenotypes similar to well-characterized probiotic strains. Molecular fingerprinting of all isolates by capillary electrophoresis of 16S-23S rRNA interspacer amplicons was coupled with chaperonin-60 universal target (*cpn*60 UT) sequencing in a subset, tentatively identifying 96% of isolates although only 19% were sequenced. Most isolates belonged to *Lactobacillus, Streptococcus, Bifidobacterium* or *Gardnerella*, with a total of 37 species in 15 genera, as well as 5 potentially novel organisms, identified in this study. This sensitivity was likely enhanced by phenotype-based selection on two chromogenic media formulations. Identification of double-strong isolates may provide a rational basis for selection and further characterization of vaginal probiotics, with potential application as part of HIV prevention initiatives in western Canada and East Africa.

## Introduction

Colonization of the female genital tract by *Lactobacillus* species is recognized as critical for overall vaginal health and resistance to infection by bacterial and viral pathogens, including HIV [1], [2], [3]. Bacterial vaginosis (BV) is a frequently asymptomatic clinical condition defined by a reduction in vaginal *Lactobacillus* populations and overgrowth of anaerobic and Gram-negative organisms [4]. Increasingly linked with negative reproductive health outcomes, such as miscarriage, premature birth, post-operative infections, pelvic inflammatory disease and HIV infection, its etiology and clinical course remain poorly defined with a consequent lack of effective prevention and treatment strategies [5], [6], [7].

Under the influence of estrogen, vaginal epithelial cells store glycogen, which is hydrolysed to glucose and metabolized by vaginal bacteria [8]. Excreted lactate, especially by *Lactobacillus* species, reduces the overall pH of the vaginal lumen to “normal” levels, in the range of 3.9 to 4.5 [9], [10]. Along with effectors such as H_2_O_2_ and bacteriocins, acid production by *Lactobacillus* is believed to discourage overgrowth of other bacterial genera including *Streptococcus*, *Gardnerella*, *Bacteroides* and *Mycoplasma* [11]. H_2_O_2_ produced by a *Lactobacillus* strain has been shown to inactivate HIV *in vitro* [12]. Production of acid and H_2_O_2_ may be synergistic, since bacterial H_2_O_2_ is more likely to remain stable at lower pH *in vitro* [13].

The ability of *Lactobacillus* to produce acid from mannose scavenged as a carbon source has also been hypothesized to block HIV infection through binding and digestion of mannosylated residues on viral glycoproteins [14] and possible subsequent inactivation through reduction of pH [15]. Interestingly, two promising molecules currently being studied as potential topical agents for blocking HIV infection (microbicides) also bind mannose residues on the viral surface [16], [17]. The application of live *Lactobacillus* organisms with potential benefit to the host (probiotics) is well-established in research and clinical practice, including in the African context [18], however the potential of this approach to block HIV infection at the mucosal surface has only begun to be explored [19].

The purpose of this study is to provide an improved strategy for selection and characterization of potentially beneficial bacteria isolated from specimens provided by a group of women with high risk of exposure to HIV. Protocols were optimized using a set of previously characterized strains and tested further through selection and characterization of vaginal isolates from Canadian adolescents. Since the specific application we propose for these organisms is as vaginal probiotics for prevention of HIV infection, we propose that identifying and artificially increasing the levels of known mannose-scavenging, HIV-inactiviting probiotics in a population may represent a novel approach to HIV prevention in high-risk groups. Little is known about vaginal microbiology in African women, particularly those who are commercial sex workers. In this study, we provide insight into the astonishing phenotypic, species and strain-level diversity in vaginal microbiota for this group.

## Results

**Figure 1:**
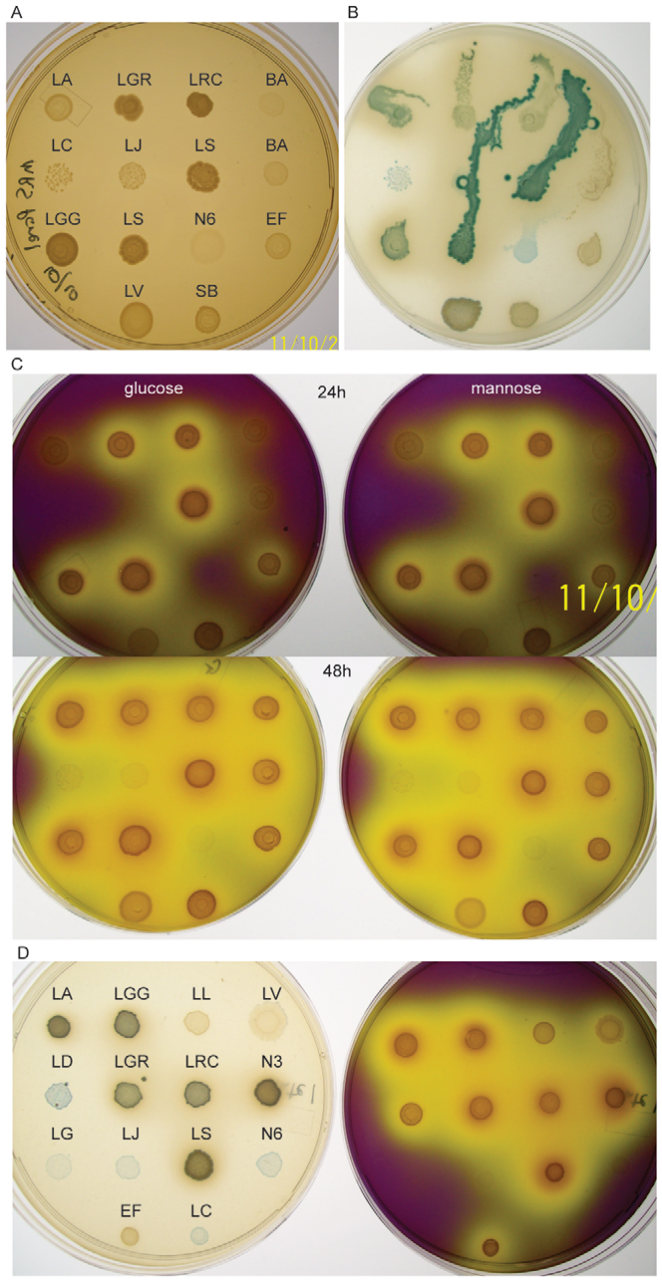
Phenotyping of panel micro-organisms. Overnight cultures of panel strains were adjusted to 0.5 McFarland and spotted onto MRS (Fig. 1A), mBH containing glucose as carboydrate source (Fig. 1B), mBG containing glucose (Fig. 1C, left) or mBM containing mannose (Fig. 1C, right) for phenotypic analysis using a replicator. Experiment was repeated on mBH2 containing glucose and mannose (Fig. 1D, left) and mBM medium (Fig. 1D, right). *E. faecalis* (EF) was chosen as positive control for acid and negative control for H_2_O_2_ production, while *L. crispatus* (LC) was chosen as positive control for H_2_O_2_ production and negative control for acid production. Key to strain identifiers: **LA**: *Lactobacillus acidophilus* 8/4, **LC**: *L. crispatus* DSMZ, **LGG**: *L. rhamnosus* GG, **LGR**: *L. rhamnosus* GR-1, **LG**: *L. gasseri* DSMZ, **LS**: *L. salivarius* AWH, **LV**: *L. vaginalis* DSMZ, **LRC**: *L. reuteri* RC-14, **N6**: *L.* sp. N6, **LD**: *L. delbrueckii* 151, **LJ**: *L. jensenii* DSMZ, **N3**: *L. salivarius* N3, **BA:** *Bifidobacterium animalis* B30, **LL**: *Lactococcus lactis* ATCC, **EF**: *Enterococcus faecalis* ATCC, **SB:** *Streptococcus bovis* ATCC. See Table 3 for full strain descriptions. Note that strain labels in A are the same for B/C, while labels on left-hand plate in D are the same for the right-hand plate.

Phenotyping experiments were optimized using a panel of relevant bacterial strains and tested for acid and H_2_O_2_ production using chromogenic media (see Methods). Qualitative differences in the intensity of yellow colour on acid-detecting medium and intensity/shade of blue colour on H_2_O_2_–detecting medium were assessed (Fig. 1). Initially, strains were inoculated by replicator onto Mann-Rogosa-Sharpe (MRS) medium for *Lactobacillus* (Fig. 1A), modified Brucella- H_2_O_2_ (mBH) medium made with commercial Brucella agar base (Fig. 1B), and a non-commercial Brucella base formulation with added glucose (mBG) or mannose (mBM) media (Fig. 1C, see Methods). Phenotypic differences in colour reaction on mBH medium were striking, with several shades of blue, green, brown and black observed (Fig. 1B). Acid production was virtually identical on mBG and mBM media, therefore study isolates were tested for acid production on mBM medium only. This experiment was repeated with an overlapping set of panel strains and a second formulation of mBH (mBH2) made with the same non-commercial base as mBM and 1∶1 glucose/mannose as substrate, with similar results observed (Fig. 1D). Interestingly, probiotic strains (*Lactobacillus* GG, GR-1, RC-14, *L. acidophilus* 8/4 and *L. salivarius* AWH) were all strong producers of acid and H_2_O_2_. *L. crispatus*, *L. gasseri* and *L. jensenii*, as well as the Nairobi isolate L6, were all acid-negative or weak producers of acid, and produced a pale blue colour indicating H_2_O_2_ production. Based on these results, *L. crispatus* was selected as positive control for H_2_O_2_ production and negative control for acid production. It should be noted that *L. crispatus* did consistently produce very pale yellow colour on test plates, and therefore should be considered a weak acid control. *E. faecalis* was selected as negative control for H_2_O_2_ production, producing solid white spots on test media and strong positive control for acid production (Fig. 1).

**Figure 2:**
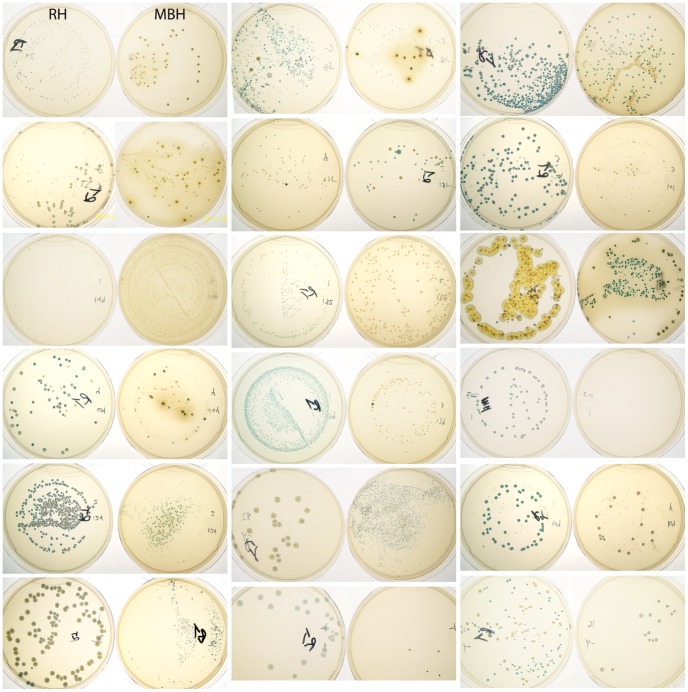
Primary isolation on chromogenic media. Vaginal samples were serially diluted and plated on RH (left) and mBH (right) media containing glucose as carbohydrate source (see Methods). One of four dilutions inoculated for each of 18 selected samples on both media are shown. A single sample did not grow on mBH (fourth sample down on right side). Note dark green and bright yellow colonies, possibly indicating elevated concentrations of H_2_O_2_ in the surrounding media.

In the pilot study, whole vaginal samples were serially diluted and plated on Rogosa-H_2_O_2_ (RH) medium (see Methods). However, growth on RH was only observed in 36 out of 48 pilot study samples. Since mBH medium has recently been reported to enhance growth and H_2_O_2_ production in a wider range of *Lactobacillus* isolates [20], it was decided to plate samples from the main study group on both RH and mBH, which resulted in recovery of isolates from all individuals. Differences in phenotype and number of colony-forming units for each sample on RH compared to mBH (Fig. 2) suggest that they provided a more complete profile of culturable organisms together than either medium on its own. When considering assortment of colony types and colour differences between colonies on both media, the profile generated for each individual is strikingly unique (Fig. 2). Several samples produced colonies with very dark blue colour and some with green or even yellow colour (eg. individual 2360, Fig. 2), possibly indicating high concentrations of H_2_O_2_ in the surrounding media [21].

**Figure 3:**
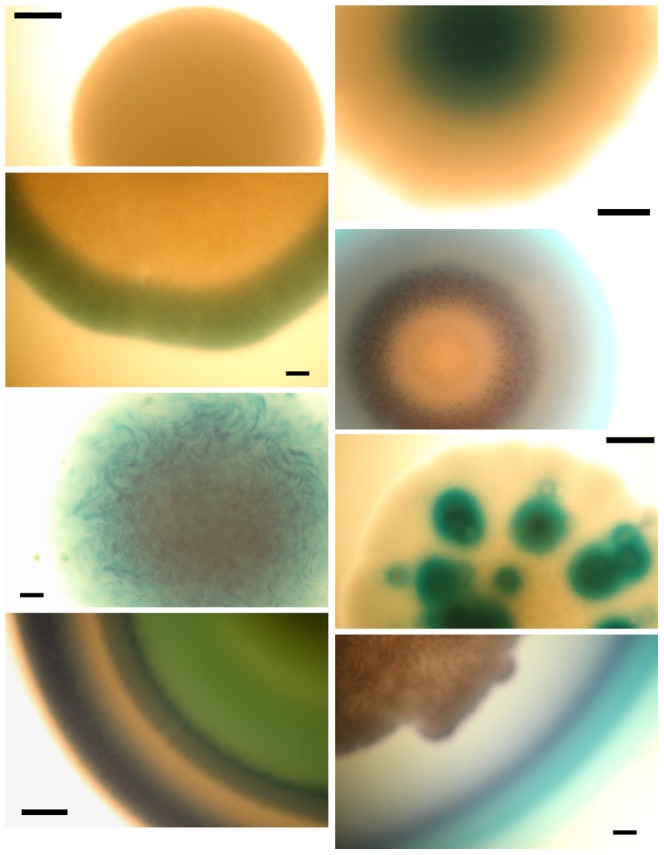
Details of colonies selected for isolates. Light micrographs of selected colony phenotypes growing on mBH2 medium with glucose and mannose as carbon sources, including H_2_O_2_-negative (top left), and various H_2_O_2_-positive phenotypes, allowing for selection of a wide diversity of phenotypes. Bar = 100 µm.

Instead of random sampling of colonies on plates, the strategy for isolate selection in this study was to pick distinct colonies based on size, colour and morphology. The major benefit of this approach compared to random selection of isolates is that a greater range of bacteria is sampled by selecting a relatively small number of isolates. The colour reaction greatly increased the number of phenotypic criteria used to select colonies, including being able to distinguish between H_2_O_2_-positive and -negative colonies, as well as variation in blue/green colour, blue-ringed vs. blue-centred colonies, blue-speckled colonies, etc. (Fig. 3). In the pilot study, a total of 192 isolates from 42 samples (16 individuals) were selected and assayed for H_2_O_2_ production on mBH medium, and for acid production on mBM medium.

**Figure 4:**
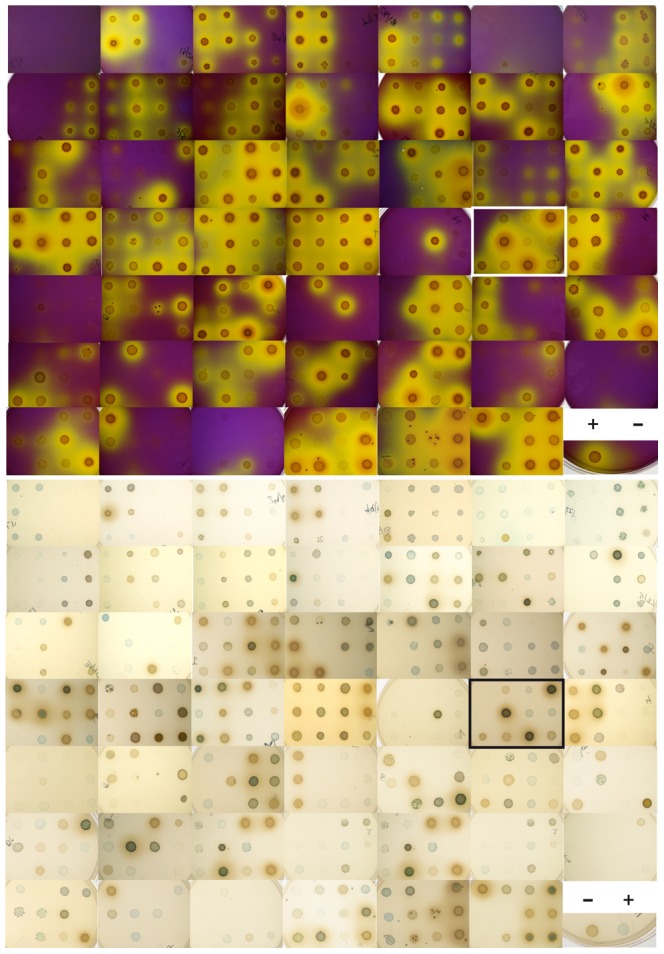
Phenotypic analysis of primary Nairobi isolates. Acid production indicated by yellow colour on mBM medium with mannose as the only carbon source (top), and H_2_O_2_ production indicated by blue colour on mBH2 medium with both glucose and mannose (bottom) for 576 isolates from 96 individuals. Note “double strong” isolates in highlighted panel, resembling phenotype of probiotic strains. Positive and negative controls were run on each plate (examples shown in bottom right-hand corner).

In phenotyping assays, brighter yellow colour was interpreted as increased acid production and darker blue/black colour was interpreted as stronger H_2_O_2_ production and scored on a 4-point scale (see Methods). Most isolates in the main study were acid- or H_2_O_2_-positive and approximately half were positive for both phenotypes (Table 1, Fig. 4). Distribution of phenotypes was approximately equal in the two study groups. A small percentage of Nairobi isolates (37/576 or 6%) were defined as “double-strong”, resembling the phenotypic profile of probiotic strains examined during optimization experiments (Table 1, Fig. 4). Based on Gram stain analysis, 22 isolates (4%) appeared to be pleiomorphic or a mixture of rods/cocci or other bacterial morphotypes, indicating that isolates picked from CFU plates did not always result in pure cultures (Table 1).

**Table 1:** Phenotypic characteristics of isolates.

	Pilot study (N = 192)	Main study (N = 576)
Acid production		
0 (no yellow)	63 (33%)	216 (38%)
1 (pale yellow)	88 (46%)	170 (30%)
2 (medium yellow)	28 (15%)	115 (20%)
3 (bright yellow)	13 (7%)	75 (13%)
H_2_O_2_ production		
0 (no blue)	83 (43%)	206 (36%)
1 (pale blue)	75 (39%)	228 (40%)
2 (medium blue)	32 (17%)	118 (20%)
3 (dark blue)	2 (1%)	24 (4%)
Acid and H_2_O_2_ production		
Double-negative (0/0)	35 (18%)	110 (19%)
Acid only (1/0, 2/0, 3/0)	48 (25%)	96 (17%)
H_2_O_2_ only (0/1, 0/2, 0/3)	28 (15%)	106 (18%)
Double positive (1/1–3/3)	81 (42%)	264 (46%)
Double-strong (2/3, 3/2, 3/3)	2 (1%)	37 (6%)
Appearance on Gram stain		
Gram-positive rods	-	337 (59%)
Gram-positive cocci	-	150 (26%)
Gram-variable coccobacilli	-	58 (10%)
Gram-negative rods	-	5 (1%)
Pleiomorphic/mixed	-	22 (4%)

**Figure 5:**
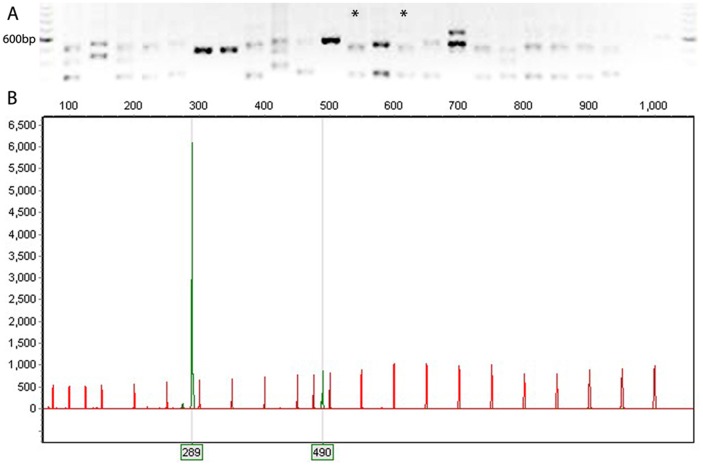
Interspacer region (ISR) amplicons and electropherograms. A. Agarose gel showing ISR amplicons for 24 isolates (two images from a single gel, spliced, inverted and adjusted for brightness and contrast only). Lanes with asterisks indicate isolates with the profile shown in B. The darker band in the ladder on either end of the gel indicates 600 bp. B. Electropherogram showing precisely sized amplicons (in green) in comparison to ROX-labelled size standard with 23 fragments from 50–1000 bp (in red). Peak indicates the strength of the fluorescent signal during electrophoresis, corresponding to the amount of amplicon of that size in the PCR. The isolate represented by this electropherogram would be interpreted as having a major peak at 289 bp and a minor peak at 490 bp.

**Figure 6:**
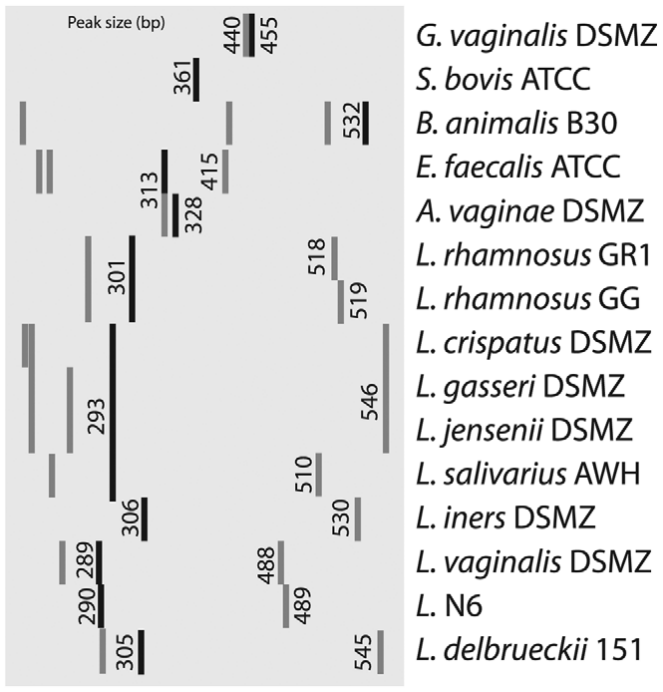
ISR profile for selected panel strains. ISR profiles for panel strains and isolates. The size (number of base pairs) for most major and minor amplicons is shown. Profiles were visualized using Java TreeView v 1.1.4r3. *L. = Lactobacillus, E. = Enterococcus, A. = Atopobium, S. = Streptococcus, G. = Gardnerella, B. = Bifidobacterium*.

Sizing of ISR amplicons by capillary electrophoresis resulted in an electropherogram of 16S-23S interspacer regions in each isolate (see Methods). Peak number and size was highly variable, creating a specific profile or fingerprint for each isolate in relation to an internal size standard (Fig. 5). The amplicon with the greatest peak height within each isolate was defined as the “major” peak, while amplicons with smaller peak heights were defined as “minor” peaks. Initially, ISR profiles were generated for 14 reference panel strains (Fig. 6). All *Lactobacillus* panel strains had a similar profile, with a major peak of 289–305 bp and a minor peak of 488–546 bp. Next, ISR profiles were generated for all pilot study and Nairobi isolates. Electropherograms were grouped according to presence of major and minor peaks in 38 groups ranging in size from 2 to 108 isolates, while 12 isolates had completely unique profiles (complete listing and description of major/minor peaks for each isolate is provided in Table S1).

**Figure 7:**
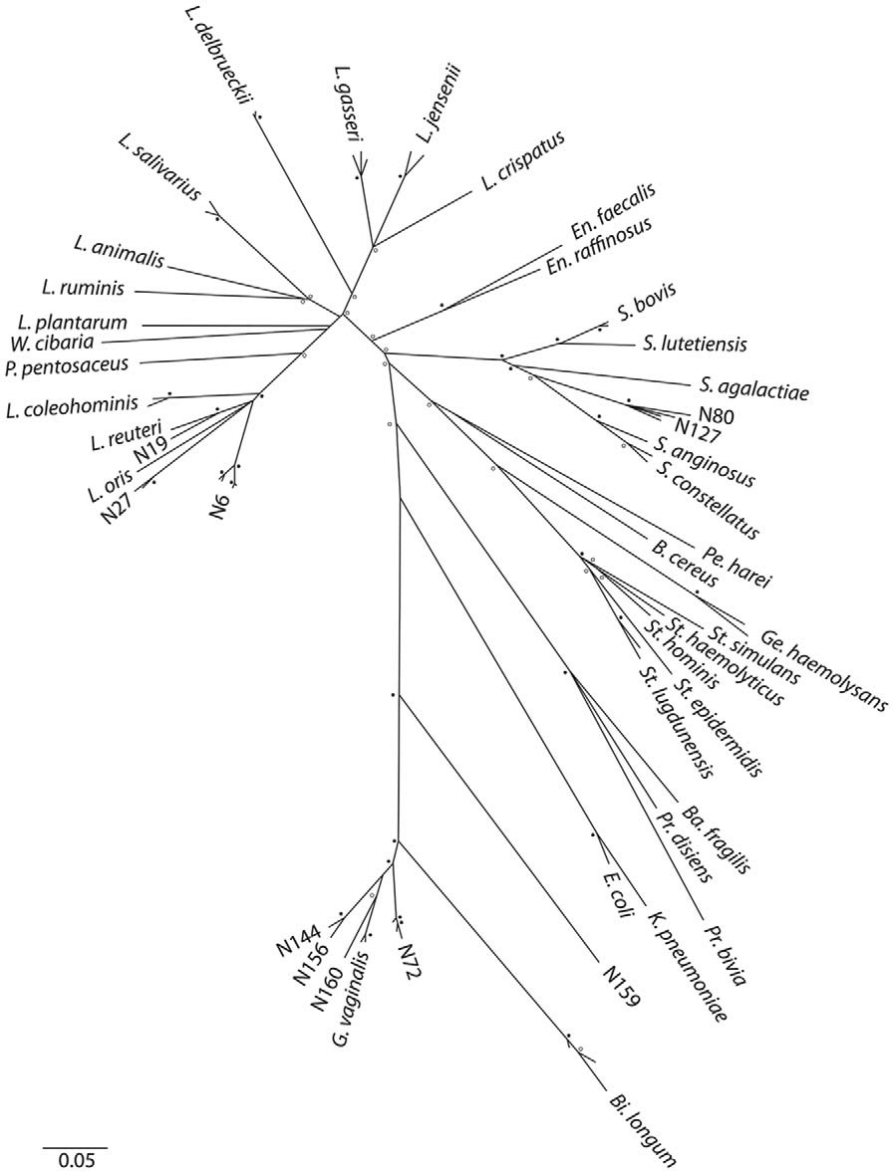
Phylogenetic relatedness of 149 isolates. Phylogeny of 149 isolates based on *cpn*60 UT sequence. Branch length indicates estimated phylogenetic distance (sum = 7.35, bar = 0.05). Bootstrap values greater than 50% are indicated with circles, based on 1000 replicates (Mega 4.0). N = Nairobi isolate, *L.* = *Lactobacillus*, *P. = Pediococcus, En. = Enterococcus, S. = Streptococcus, B.* = *Bacillus, Ge. = Gemella, St. = Staphylococcus, Ba. = Bacteroides, Pr.* = *Prevotella*, *E.* = *Escherichia*, *K.* = *Klebsiella*, *Bi. = Bifidobacterium, G*. = *Gardnerella*.

A total of 149 isolates were selected for full-length *cpn*60 UT sequencing and identification by comparison with cpnDB (nearest neighbour and percent identity for each sequenced isolate is detailed in Table S1), including all “double-strong” isolates and representatives from all groups based on ISR profile. The phylogeny of sequenced isolates was consistent with current knowledge of culturable vaginal microbiota (Fig. 7). Unsurprisingly, genera in order Lactobacillales are well-represented (97/149 sequenced isolates). However, strains from a number of genera representing four phyla were isolated in this study, including other Firmicutes (*Staphylococcus*, *Bacillus*, *Gemella*; 15 isolates), Actinobacteria (*Gardnerella*, *Bifidobacterium*; 28 isolates), Bacteroidetes (*Bacteroides, Prevotella*; 5 isolates), and Proteobacteria (*Escherichia, Klebsiella*; 4 isolates). Several potentially novel groups of vaginal bacteria were observed, including isolates similar to the N6 strain isolated in preliminary studies. This group has several branches with robust nodes, and a *cpn*60 UT sequence about 90% similar to both *L. vaginalis* and *L. reuteri* reference strains. Several other *L. reuteri*-related isolates distinct from *L.* N6 were also observed, including one from a pilot study sample, identified as *L. oris*. Extensive sequence diversity was observed for *G. vaginalis* isolates, with the majority less than 90% identical to the type strain.

**Figure 8:**
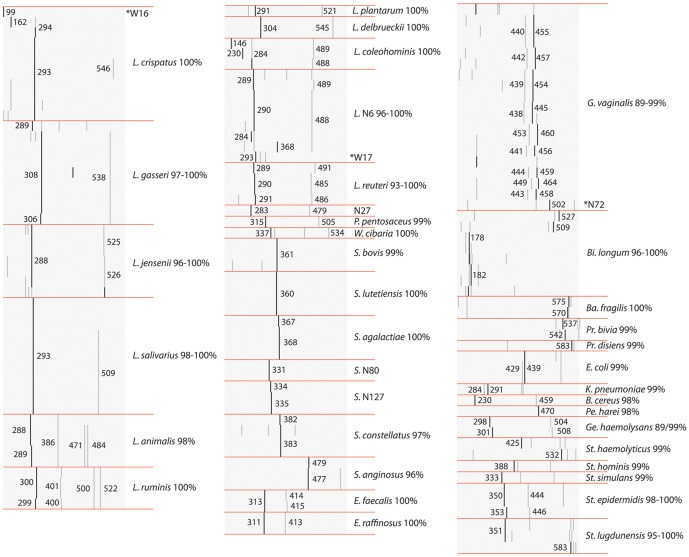
Comparing cpn60 UT sequences and ISR profiles of cultured isolates. Species identification of isolates by *cpn*60 UT sequencing is shown alongside ISR profile for each isolate (grey bars), demonstrating correspondence between the two targets. Black bands indicate major peaks and grey bands indicate minor peaks. Image generated using Java TreeView v 1.1.4r3. Abbreviations of genera as in previous figure.

A comparison of *cpn*60 UT sequence and ISR profile in a subset of isolates suggests that these targets co-vary across organisms from both study groups (Fig. 8). Isolates identified as *L. crispatus*, *L. gasseri* and *L. jensenii* by *cpn*60 sequencing all have distinct ISR profiles, with major bands at 293, 308 and 288 bp respectively. All *L. salivarius* isolates also had a major peak at 293 bp but most could be distinguished from *L. crispatus* by the presence of a minor peak at 509 bp. In this study, all isolates with a major peak at 293bp and no *L. crispatus*-like minor peak at 546 bp were sequenced and determined to be *L. crispatus* (see Table S1). Isolates in the *L.* N6/*L. reuteri* groups had peak profiles resembling the *L. vaginalis* reference strain, with a major peak around 290 bp and a minor peak around 488 bp. In contrast to *Lactobacillus*, most isolates identified as *Streptococcus* had no minor peaks. Major peaks only a few base pairs apart were consistently associated with unique *cpn*60 UT sequences (eg. *S. bovis, S. lutetiensis, S. agalactiae*). In parallel to observed *cpn*60 UT sequence diversity, *G. vaginalis* isolates had related but divergent ISR profiles. The *G. vaginalis* reference strain had major/minor peaks at 455/440 bp, but other isolates had dual peaks that “wobbled” higher and lower, while always maintaining an interval of 15 bp. Another example of this was observed in two isolates from a single individual, one of which was 99% identical to *Gemella haemolysans* and had major/minor peaks at 298/504 bp, and the other only 89% identical to *G. haemolysans* that had peaks 3 bp larger at 301/507 bp. There were only three cases of an obvious discrepancy between *cpn*60 UT sequence and ISR profile (marked with asterisks in Fig. 8), indicating the presence of more than one strain in these “isolates”. The identity of all unsequenced isolates was subsequently inferred based on similarity of ISR profile to sequenced isolates. In addition to 4 isolates with failed electropherograms, 22 isolates (2.9%) belonged to ISR groups with no sequenced representatives. This approach resulted in inferred strain-level identification of 97% of isolates by ISR profile after *cpn*60 UT sequencing of only19% of isolates.

Phenotype and phylogeny of all 768 isolates in this study is summarized in Table 2. Overall, a striking similarity in major groups of organisms was observed in these socially and geographically distant groups of women. Although a greater number of distinct phylotypes was observed among East Africans, this may be due to three times as many isolates being chosen from six times as many women in this group. Well-known vaginal organisms such as *L. crispatus, L. gasseri* and *L. jensenii*, as well as less well-characterized organisms such as N6 and *L. coleohominis* groups, all had predominantly weak acid and H_2_O_2_ production, as observed with panel strains. In contrast, *L. salivarius*, *L. animalis*, *L. ruminis* and *L. delbrueckii* were predominantly strong acid producers and most “double-strong” isolates were observed in these groups. The double-strong phenotype was also observed in *Streptococcus*, *Pediococcus, Weissella* and *Bifidobacterium* isolates.

**Table 2:** Summary of isolates.

		Study^1^		Summary of ISR (bp)^2^		Phenotype^3^		
cpn60 phylotype	Isolates (#)	Pilot (#)	Main (#)	Major Mode (range)	Minor Mode (range)	Acid (Mode Score)	H_2_O_2_ (Mode Score)	Double-strong (#)
*L.* sp. N6	108	12	96	290 (289–90)	488 (487–91)	0	1	2
*L.* sp. N27	24	7	17	283	479 (479–80)	2	0–1	
*L. oris*	1	1	0	291	183	2	2	
*L.* sp. N19	2	2	0	290	485	2	2	
*L. reuteri*	11	2	9	289	491	1	2	1
*L. coleohominis*	30	0	30	284	488 (488–9)	0	1	
*P. pentosaceus*	3	0	3	315	505	3	2	3
*W. cibaria*	1	0	1	337	356	2	2	
*L. plantarum*	1	0	1	291	521	3	1	
*L. ruminis*	6	0	6	300 (299–300)	400–401	3	2	3
*L. animalis*	24	0	24	288 (288–9)	386	3	0	5
*L. salivarius*	30	4	26	293	509 (509–10)	3	1	10
*L. delbrueckii*	3	0	3	304	545	3	3	2
*L. gasseri*	59	32	27	308 (306–8)	538 (538–9)	1	1	1
*L. jensenii*	62	30	32	288	525 (525–6)	1	1	
*L. crispatus*	108	61	47	293 (293–4)	546 (546–7)	0	1	
*E. faecalis*	8	1	7	313	415 (414–5)	2	1	
*E. raffinosus*	2	0	2	311	413	2	3	2
*S. bovis*	17	0	17	361		2	2	4
*S. lutetiensis*	20	6	14	360		1	0	1
*S. agalactiae*	31	0	31	368 (367–8)		2	2	
*S.* sp. N80	16	0	16	331		1	1	
*S.* sp. N127	11	1	10	335 (334–6)		1	1	
*S. anginosus*	5	0	5	477 (477–9)		2	2	
*S. constellatus*	32	7	25	383 (382–3)		2	2	
*Pe. harei*	1	0	1	470		1	1	
*B. cereus*	1	0	1	230	459	1	1	
*Ge. haemolysans*	2	0	2	298–301	504–8	1	2	
*St. simulans*	1	0	1	333	424	0	1	
*St. haemolyticus*	2	0	2	425		1	0	
*St. hominis*	1	0	1	388		1	0	
*St. epidermidis*	12	4	8	350–3	444 (444–6)	1	0	
*St. lugdunensis*	3	0	3	351		1	0	
*St. pisciferment.*	1	0	1			2	0	
*Pr. bivia*	2	0	2	537–43	771–74	1	0	
*Pr. disiens*	1	0	1	583	354	1	0	
*Ba. fragilis*	2	0	2	570–5		1	0	
*E. coli*	5	0	5	439	429	1	0	
*K. pneumoniae*	1	0	1	291	435	1	0	
N159	1	0	1	397		1	0	
*Bi. longum*	14	6	8	177–82		2	0	
*Bi. catenulatum*	9	0	9	530 (527–32)	227	3	0	4
*G. vaginalis*	68	5	63	459 (438–466)	441 (438–460)	0	0	1

1 Group based on nearest neighbour in *cpn*60 reference database, *L. Lactobacillus, P. Pediococcus, W. Weissella, E. Enterococcus, S. Streptocococus, Pe. Peptoniphila, B. Bacillus, Ge. Gemella, St. Staphylococcus, Pr. Prevotella, Ba. Bacteroides, E. Escherichia, K. Klebsiella, Bi. Bifidobacterium, G. Gardnerella*.

2 Interspacer size in base pairs for major and first minor peak. Value shown is the mode interspacer size with range of observed values shown in brackets.

3 Phenotypic information summarized as mode values of each *cpn*60 group in terms of intensity of acid and H_2_O_2_ production on 4-point scales (see Methods), and total number of double-strong isolates in each *cpn*60 group.

## Discussion

Initial studies using reference strains indicate that well-characterized probiotics are strongly acid- and H_2_O_2_-positive on test media, suggesting that these characteristics may be related to the exertion of beneficial effects *in vivo*. Interestingly, *Lactobacillus* GR-1 and RC-14 have not previously been shown to be H_2_O_2_-positive (G. Reid, personal communication). Our result is likely due to culture of these organisms on the recently described mBH medium, which was developed to enhance H_2_O_2_ production in a wide variety of *Lactobacillus* organisms [20]. Most probiotic identification strategies involve isolation of *Lactobacillus* colonies on Rogosa media without chromogen and then testing these isolates for H_2_O_2_ production on Rogosa with chromogen [20], [22], [23]. In this study, primary isolation was carried out on media with H_2_O_2_-sensitive chromogens, allowing for colonies to be selected based on colour patterns, greatly increasing the number of different types of colonies that could be distinguished by simple observation. Limitations of this approach include an unequal number of isolates selected from each sample and a lack of quantitative information about the proportion of total vaginal bacteria represented by each phenotype. Phenotypic variability on both RH and mBH media was striking. Although the scope of this study is qualitative and reproducibility of these results has not been determined, these observations reinforce the concept that phenotypic profiles of culturable *Lactobacillus* are highly idiosyncratic. This is consistent with observations of unique individual-level species/strain in culture-independent deep sequencing profiles of vaginal microbiota previously reported in this group [24].

This study has resulted in a unique dataset of phenotypic data related to acid and H_2_O_2_ production, however the biological significance of striking colour differences between organisms on H_2_O_2_-detecting media has not been fully defined. Variation in shade from blue to green and yellow zones surrounding colonies may be attributed to the different-coloured oxidation products of TMB by HRP in the presence of H_2_O_2_ (blue, yellow or a mixture of the two producing green). At higher H_2_O_2_ concentrations, the blue product is either partially degraded, resulting in green colour, or completely degraded, resulting in yellow colour [21]. In phenotyping assays, no yellow was seen, however culture spots ranged from green to pale blue, dark blue, brown and black and were scored based on colour intensity/darkness, however whether darker colours are due to higher H_2_O_2_ concentrations was not tested in this study. Whether *L. crispatus*, pale blue in appearance, is an appropriate positive control for dark black *L. salivarius* strains, for example, has not been resolved. However, the consistent appearance of chosen controls on every plate of isolates tested does indicate stability of phenotype and media formulation over all experiments. The biological significance of colour variation in primary isolates can only be speculated about based on current data, however the large number of phenotypically and phylogenetically distinct organisms isolated in this study can be attributed to the utility of qualitative differences in blue colour as a basis for strain selection. To our knowledge, the surprising diversity of H_2_O_2_-related phenotypes on primary isolation, including elaborately coloured ring structures and halo-like effects, has not been described previously. Ringed patterns suggest coordinated gene expression within colonies, possibly linked to quorum-sensing mechanisms, while speckled patterns suggest phage-mediated lysis or autolysis with subsequent release of H_2_O_2_. Further work will be required to elucidate regulation of H_2_O_2_ production in vaginal isolates and explain these unique observations.

A further limitation of phenotypic assays also mitigating interpretation of intensity data in acid-producing strains is that colour is strongest in strains that grow to highest density on test media, not necessarily reflecting *in vivo* characteristics. Our results indicate that some strains grow very strongly on mannose-containing media and produce low pH in surrounding media that is consistent with normal vaginal acidity (pH∼4) [9]. Although the possibility was not addressed directly in this study, mannose scavenging coupled with synergistic production of acid and H_2_O_2_ [13] may represent a co-evolved antiviral role for lactic acid bacteria in the vaginal microbiota.

Gram stain analysis revealed that some “isolates” are likely more accurately described as “enrichments”, consisting of one or more bacterial strains. Phenotypic data can be considered to validly represent an element that has been isolated from the microbiota, whether or not this element consists of one or more bacterial strains. Interspecific interactions in enrichments may result in differential acid and H_2_O_2_ production, with implications for defining probiotic effects *in vivo*, however further work will be required to determine phenotypic regulation in enrichments.

This is the first study to combine analysis of 16S-23S rRNA interspacer profiles with *cpn60* UT sequence for molecular identification of a large group of isolates. The 60 kDa chaperonin or heat shock protein (also known as HSP60 and GroEL), is increasingly used for identification of isolates and to characterize complex microbial communities [25]. A total of 37 previously described species in 15 genera were detected in this study, as well as 6 well-defined groups with sequences <97% similarity to anything in cpnDB, the *cpn*60 sequence database (www.cpndb.ca). Our ability to resolve species diversity in genera of interest, including 14 *Lactobacillus*, 9 *Streptococcus* and 6 *Staphylococcus* species, as well as extensive genetic variability in *Gardnerella* isolates, can be attributed to the increased resolution of the *cpn60* UT, as we have previously shown in culture-independent studies [24]. Close correspondence between *cpn*60 UT and ISR was observed in a subset of 149 strains for which both targets were characterized. Although further studies will be required to validate inferences in identity made based on ISR profile alone, our results indicate that capillary electrophoresis of amplicon from the ISR may be efficient and accurate, comparing favourably with Sanger sequencing for phylogenetic characterization of isolates. Further, variability in *cpn*60 UT sequence appears to correspond to variability observed in ISR profile, most strikingly for *Gardnerella*-related isolates. An understanding of the possible biological significance of these differences will require further investigation.

As previously mentioned, it is likely that a number of isolates are in fact mixtures of strains (enrichments). Many minor peaks were seen in ISR profiles of one *cpn*60 UT sequence group that were consistent with other *cpn*60 UT sequence groups. For example, a *Prevotella disiens* isolate resembled Gram+ cocci, and had peaks resembling other *Prevotella* isolates, as well as a peak characteristic of *S. epidermidis*. In other cases where isolates had Gram stain characteristics inconsistent with molecular profile, the *cpn*60 UT sequence and ISR profile were always consistent with each other. Since DNA for molecular analysis was harvested from isolation plates prior to phenotyping, and Gram stained slides were prepared after incubation of phenotyping plates 48 h later, it is likely that strains present at lower concentration at the time of DNA isolation may have overgrown the isolate during subsequent incubation. Therefore, an important limitation of this study is that phenotypic profile cannot be tied with certainty to phylogenetic profile.

Only a small number of studies have addressed in-depth molecular profiling of vaginal microbiota in African women [24], [26]. Culture-based studies provide a view of microbiota that is distinct from that generated by deep sequencing, since they provide phenotypic as well as genotypic data despite the limitations imposed by the selective effects of culture media. Furthermore, when selective media are employed, sensitivity of detection can be even greater than that provided by deep sequencing. We observed several *Lactobacillus* phylotypes only on selective media and not by culture-independent approaches in a subset of samples [24]. This finding indicates the importance of culture-based approaches for complete understanding of the phylogenetic structure of bacterial groups of interest. For example, *L. gasseri* was not observed by deep sequencing although it is a common species in the vagina and well-represented among cultured isolates. This suggests either that the actual quantitative distribution of *L. gasseri* is below the limit of detection for deep sequencing, or that some unknown factor biases its representation by deep sequencing. The observation that a small number of deep sequencing reads were detected for several other *Lactobacillus* phylotypes indicates that the limit of detection for culture on *Lactobacillus*-selective media is lower than the limit of detection for deep sequencing. This is also true for *Streptococcus* and *Staphylococcus* species, with only a very small number of sequences and greatly reduced number of species detected by deep sequencing compared to culture [24].

In conclusion, we have described a novel combination of methods for culture-based selection, evaluation and identification of a large number of isolated bacteria from vaginal swab samples. The principal advantage of our strategy is the ability to distinguish unique colour patterns in organisms upon isolation, minimizing repeated isolation of organisms which would otherwise have a similar appearance. Phenotyping assays resulted in the identification of a small number of “double-strong” acid- and H_2_O_2_-producing isolates resembling the phenotype of known probiotics such as *Lactobacillus* GG, GR-1 and RC-1. Our identification strategy was based on comparisons of two independent molecular targets, and greatly reduced the number of sequences required for tentative identification of all isolates. Further work will be required to confirm our finding of excellent correspondence between ISR profile and *cpn*60 UT sequences for isolate identification.

Whether our observations have implications for competitive exclusion of bacterial vaginosis-associated bacteria and/or possible blocking of HIV infection through binding and inactivation of viral particles *in vivo* has yet to be determined. Application of vaginal probiotics for protection against sexually transmitted diseases and HIV infection remains largely conceptual [19]. Whether or not the ecology of vaginal bacteria might be manipulated through the application of probiotics to improve vaginal health and reduce susceptibility to infection is the subject of future studies. This study provides one of the first overviews of the astonishing species and strain diversity of culturable vaginal microbiota in African commercial sex workers, while reinforcing the surprising uniformity of the most frequently observed vaginal organisms across all groups of women studied around the world to date.

## Methods

### Ethics statement

All study procedures were approved by the Ethics Review Boards of the University of Manitoba and the University of Nairobi.

### Study groups

Self-collected vaginal samples and a self-reported written questionnaire regarding age, current sexual activity and birth control use were provided by young women attending a gynecology clinic in inner-city Winnipeg (Jun.–Aug. 2005), as previously described [27]. A subset of 42 samples from 16 individuals were selected for in-depth culture-based analyses (see Table S2 for characteristics of samples and participants). In Nairobi, mid-vaginal swabs were collected by a single physician during routine research visits as part of a well-established collaborative study for STI/HIV prevention in the Departments of Medical Microbiology at the University of Manitoba and University of Nairobi as previously described [28], [29]. Data from interviews and clinical examination were also collected by study personnel for each individual. A single sample from a subset of 96 individuals was selected for culture-based analyses (see Table S3 for participant characteristics).

### Sample collection, processing and BV diagnosis

For both studies, samples were collected using double-headed devices without buffer (Starplex Scientific, Inc., Etobicoke), as previously described [27], [30] with swab devices collected by study personnel, delivered to the laboratory and processed within three hours of sample collection. In Winnipeg, one swab was used to prepare a smear for Gram stain analysis, while the other was snipped into 1 ml sterile RTF-glycerol buffer (0.045% w/v potassium phosphate monobasic, 0.045% w/v potassium phosphate dibasic, 0.09% w/v sodium chloride, 0.018% w/v magnesium sulphate, 0.038% w/v EDTA, 0.04% w/v sodium bicarbonate, 0.02% dithiothreitol and 10% v/v glycerol) [31] in 1.5 ml Eppendorf tubes and stored at −80°C until culture. In Nairobi, one swab head was immediately frozen as described above and shipped to Winnipeg in liquid nitrogen for culture-based analyses, with Gram stained slides prepared by clinic personnel from a separate swab. BV was diagnosed by examining Gram stained vaginal swab smears and applying the criteria of Hay and Ison (for Winnipeg samples) [32] or Nugent (for Nairobi samples) [33] as previously described [24], [27]. Both scoring systems result in a diagnosis of BV−, BV-intermediate or BV+ based on assessment of proportion of *Lactobacillus* morphotypes vs. Gram-negative or variable and/or curved rods.

### Quantitative culture and strain isolation

The focus of this study was to isolate vaginal lactic acid bacteria (LAB), therefore *Lactobacillus*-selective Rogosa medium (without chromogen) and Rogosa-H_2_O_2_ (RH) medium (with H_2_O_2_-detecting chromogens, see below) was used for strain isolation in the Winnipeg study. Nairobi samples were cultured on Rogosa, RH and a recently described medium called modified Brucella H_2_O_2_ (mBH) medium, recently reported to support growth and H_2_O_2_ production in a wider range of *Lactobacillus* strains. Frozen swabs were thawed, vortexed vigorously, opened in a biosafety cabinet to prevent contamination, and squeezed against the side of the tube prior to being discarded. Eluted sample was centrifuged and resuspended in 1 ml PBS. Decimal dilutions were prepared and a 50 µl aliquot of each dilution spread-plated on 1 plate each of 1) Rogosa; Rogosa medium (Difco) prepared according to manufacturer's instructions without chromogenic reagents, 2) RH; Rogosa medium with (per litre) 1 ml 1% w/v horseradish peroxidase (HRP; Sigma) and 250 mg tetramethylbenzidine (TMB; Sigma) for chromogenic detection of H_2_O_2_, and 3) mBH medium (per litre: 41 g Brucella agar (EMD), 20 g soluble starch (Becton-Dickinson, 0.86 g magnesium sulfate anhydrous (Sigma), 0.18 g manganese sulfate monohydrate (Sigma), 1 ml 5% w/v hemin (Sigma) dissolved in 1N NaOH (Sigma), 2.5 ml 0.5% v/v Vitamin K (Sigma) dissolved in 95% EtOH, and 50 ml horse serum (Sigma)) with chromogenic reagents as above.

Plates were incubated at 37°C for 48 h in an anaerobic chamber. RH and mBH plates were used for strain isolation by selecting colonies at dilution levels with well-separated colonies having a unique appearance as described above. Up to 10 isolates were picked per sample, sub-cultured to Mann-Rogosa-Sharpe agar (MRS; EMD) for isolates from RH or to mB (same as mBH but without chromogenic reagents) for isolates from mBH and incubated in an anaerobic chamber at 37°C for 48 h.

### Phenotypic characterization of isolates

All phenotypic assays were optimized and control strains selected using relevant organisms selected from culture collections, probiotic strains, and Nairobi isolates from preliminary studies (Table 3). Since an objective of this study was to determine the ability of vaginal LAB isolates to ferment mannose and commercial Brucella agar contains glucose in its formulation, test medium was prepared with a non-commercial Brucella base and added glucose (mBG medium) or added mannose (mBM medium), per litre: 10 g meat peptone (Becton-Dickinson), 10 g tryptone from casein (Becton-Dickinson), 2 g yeast extract (Becton-Dickinson), 5 g sodium chloride (Sigma), 15 g agar (Becton-Dickinson), 25 g mannose or glucose, as well as 0.15 g bromocresol purple (BCP) to detect acid production by whole samples and soluble starch, magnesium sulfate, manganese sulfate, hemin, Vitamin K and horse serum as for mBH.

**Table 3:** Panel of reference organisms.

Organism	Strain^1^	Media^2^	Source^3^
*Lactobacillus crispatus*	DSMZ 20584	MRS	DSMZ
*Lactobacillus gasseri*	DSMZ 20243	MRS	DSMZ
*Lactobacillus iners*	DSMZ 13335	CBA	DSMZ
*Lactobacillus jensenii*	DSMZ 20557	MRS	DSMZ
*Lactobacillus vaginalis*	DSMZ 05837	MRS	DSMZ
*Atopobium vaginae*	DSMZ 15829	CBA	DSMZ
*Streptococcus bovis*	ATCC 49147	MRS	M. Alfa, SBGH
*Gardnerella vaginalis*	ATCC 14018	CA	M. Alfa, SBGH
*Enterococcus faecalis*	ATCC 29212	MRS	W. Smoragiewicz, UQAM
*Lactococcus lactis*	ATCC 11454	MRS	W. Smoragiewicz, UQAM
*Lactobacillus acidophilus*	8/4	MRS	W. Smoragiewicz, UQAM
*Lactobacillus delbrueckii*	151	MRS	W. Smoragiewicz, UQAM
*Lactobacillus salivarius*	AWH	MRS	W. Smoragiewicz, UQAM
*Bifidobacterium animalis*	B30	MRS	W. Smoragiewicz, UQAM
*Lactobacillus rhamnosus*	GG	MRS	G. Reid, UWO
*Lactobacilllus rhamnosus*	GR-1	MRS	G. Reid, UWO
*Lactobacillus reuteri*	RC-14	MRS	G. Reid, UWO
*Lactobacillus sp.*	N6	MRS	S. Iqbal, UM
*Lactobacillus salivarius*	N3	MRS	This study

1 DSMZ: German culture collection, ATCC: American Type Culture Collection,

2 Media for routine culture; MRS: Mann-Rogosa Sharpe (EMD), CBA: Columbia Blood Agar (EMD), CA: Chocolate Agar,

3 Strains purchased from DSMZ or kindly provided by the individual named; UQAM: Universite du Quebec a Montreal, SBGH: Clinical Microbiology Lab, St. Boniface General Hospital, HSC: Clinical Microbiology Lab. Health Sciences Centre, UM: University of Manitoba, UWO: Lawson Health Research Institute, University of Western Ontario.

For isolates, growth from isolation plates was washed off with 1 ml PBS and adjusted to 0.5 McFarland in 3 ml PBS. Aliquots were transferred to sterile replicators and spotted onto MRS, mB mBM and mBH, version 2 (mBH2), as for mBH but prepared with non-commercial base (described above), 12.5 g D+mannose (Sigma), 12.5 g D+glucose (Sigma). Plates were incubated 37°C for 48 h in an anaerobic chamber. Once removed from the incubator, colour was allowed to develop for 30 min. prior to scoring and photographing of plates. H_2_O_2_ production on mBH2 was scored on a 4-point scale based on intensity of blue colour (0 = no colour, 1 = pale blue, 2 = medium blue, 3 = dark blue). Acid production on mBM was scored on a 4-point scale based on intensity of yellow colour (0 = no yellow, 1 = pale yellow, 2 = medium yellow, 3 = bright yellow). *L. crispatus* DSM20584 and *Enterococcus faecalis* ATCC 29212 were used as positive and negative controls (see Figure 1), and were cultured simultaneously on every plate used for chromogenic determination of acid and H_2_O_2_ production. A Gram stain of each isolate on MRS or mB was prepared and photographed using standard procedures, and an aliquot stored at −80°C in an equal volume of 2X freezing media (4% w/v skim milk, 1% D+ glucose and 20% v/v glycerol).

### Ribosomal interspacer analysis of cultured isolates

16S-23S rDNA interspacer (ISR) profiles were generated for reference strains, followed by isolates from Winnipeg and Nairobi. DNA was isolated from aliquots of isolate cells using Instagene Matrix (BioRad), according to the directions of the manufacturer. Briefly, a 200 µl aliquot of washed sample in PBS was centrifuged, the supernatant discarded, and the pellet resuspended in 200 µl Instagene Matrix. The suspension was vortexed, incubated at 56°C for 30 min., vortexed, incubated at 100°C for 8 min., vortexed and centrifuged. Supernatant containing isolate DNA was used for subsequent molecular analyses. Amplification of the ISR was carried out using published primers and procedures [34]. Briefly, PCR was carried out using forward and reverse primers targeted to positions near the end of the 16S rRNA gene and beginning of the 23S rRNA gene respectively (ITS-F: GTC GTA ACA AGG TAG CCG TA and ITS/R: GCC AAG GCA TCC ACC) [34]. PCR was carried out with 2 µl template DNA in a reaction mixture containing 5 µl 10× PCR buffer, 2.5 µl 50 mM MgCl_2_, 1 µl 10 mM dNTP mixture, 1 µl 25 µM each primer and 0.5 µl Taq polymerase (Invitrogen, Burlington, ON) and water to a final reaction volume of 50 µl. Cycling conditions were as follows: 95°C/2 min., followed by 40 cycles of 95°C/45 sec., 55°C/1 min., 72°C/2 min., and a final extension of 72°C for 7 min. Resulting fragments include 99 bp from 16S rDNA and 22 bp from 23S rDNA.

In order to size the ISR precisely, the 5′ end of the reverse primer was modified with the phosphoramidite dye HEX (6-carboxy-1,4-dichloro-2′,4′,5′,7′-tetra-chlorofluorescein) for fragment detection by capillary electrophoresis in an ABI 3100 sequencer. Amplicons were purified using an automated MagnaPure system (DNA Core, National Microbiology Laboratory), diluted 20-fold in water, and 2 µl mixed with 15 µl HiDi formamide (ABI) and 3 µl X-Rhodamine (ROX)-labelled MapMarker 1000 size standard (Bioventures), with 23 fragments covering a range of 50–1000 bp. The reaction mixture was denatured 5 min. at 95°C, and immediately placed on ice until electrophoresis. Amplicon reactions were run on the ABI 3100 genetic analyzer using POP7 polymer and standard denaturing electrophoresis settings for approximately 1.5 h. The resulting electropherograms were processed and amplicons sized in relation to the internal size standard using GeneMarker (SoftGenetics, State College, PA). Results were visualized using Java TreeView v1.1.4r3 (http://jtreeview.sourceforge.net, created by Alok Saldanha) [35] and representatives selected for species identification by sequencing of the *cpn*60 universal target (*cpn*60 UT).

### *cpn*60 UT-based sequencing and identification of isolates

The *cpn*60 UT region was used for isolate identification, based on published primers and procedures [36]. Briefly, target was amplified from isolate DNA using the degenerate primers H729-F: **CGC CAG GGT TTT CCC AGT CAC GAC** GAI III GCI GGI GAY GGI ACI AC and H730-R: **AGC GGA TAA CAA TTT CAC ACA GGA** YKI YKI TCI CCR AAI CCI GGI GCY TT incorporating the M13 (−40)F and M13 (48)R sequencing primer landing sites (in bold). PCR was carried out with 2 µl template DNA in a reaction mixture containing 5 µl 10× PCR buffer, 2.5 µl 50 mM MgCl_2_, 1 µl 10 µM dNTP mixture, 1 µl 25 µM each primer, 0.5 µl Taq polymerase (Invitrogen) and water to a final reaction volume of 50 µl. Cycling conditions were as follows: 95°C for 2 min., followed by 40 cycles of 95°C/30 sec., 42°C/30 sec., 72°C/30 sec., and a final extension of 2 min. at 72°C. Amplicons were purified using an automated MagnaPure system and sequenced in both directions using an ABI3700 genetic analyzer (DNA Core, National Microbiology Laboratory). Resulting sequences were assembled and trimmed using Lasergene 8 software (DNAStar, Madison, WI). Full-length UT (∼552 bp) were compared to cpnDB to determine nearest neighbour using FASTA queries [37]. A phylogenetic tree of resulting sequences was constructed in MEGA 4.0 using the Neighbour-Joining Method, as previously described [24].

## Supporting Information

Table S1 **Complete list and phylogenetic/phenotypic details of isolates.**
(PDF)

Table S2 **Characteristics of individuals providing vaginal samples in Winnipeg (N = 16).**
(PDF)

Table S3 **Characteristics of individuals providing vaginal samples in Nairobi (N = 96).**
(PDF)

## References

[1] Anukam KC, Osazuwa EO, Ahonkhai I, Reid G (2006). Lactobacillus vaginal microbiota of women attending a reproductive health care service in Benin city, Nigeria.. Sex Transm Dis.

[2] Beigi RH, Wiesenfeld HC, Hillier SL, Straw T, Krohn MA (2005). Factors associated with absence of H2O2-producing Lactobacillus among women with bacterial vaginosis.. J Infect Dis.

[3] Roberton AM, Wiggins R, Horner PJ, Greenwood R, Crowley T (2005). A novel bacterial mucinase, glycosulfatase, is associated with bacterial vaginosis.. J Clin Microbiol.

[4] Westrom L, Evaldson G, Holmes KK (1984). Taxonomy of vaginosis: bacterial vaginosis - a definition.. Scand J Urol Nephrol.

[5] Forsum U, Hallen A, Larsson PG (2005). Bacterial vaginosis–a laboratory and clinical diagnostics enigma.. Apmis.

[6] Forsum U, Holst E, Larsson PG, Vasquez A, Jakobsson T (2005). Bacterial vaginosis–a microbiological and immunological enigma.. Apmis.

[7] Larsson PG, Bergstrom M, Forsum U, Jacobsson B, Strand A (2005). Bacterial vaginosis. Transmission, role in genital tract infection and pregnancy outcome: an enigma.. Apmis.

[8] Hillier SL (1998). The vaginal microbial ecosystem and resistance to HIV.. AIDS Res Hum Retroviruses.

[9] Boskey ER, Cone RA, Whaley KJ, Moench TR (2001). Origins of vaginal acidity: high D/L lactate ratio is consistent with bacteria being the primary source.. Hum Reprod.

[10] Boskey ER, Telsch KM, Whaley KJ, Moench TR, Cone RA (1999). Acid production by vaginal flora in vitro is consistent with the rate and extent of vaginal acidification.. Infect Immun.

[11] Ronnqvist PD, Forsgren-Brusk UB, Grahn-Hakansson EE (2006). Lactobacilli in the female genital tract in relation to other genital microbes and vaginal pH.. Acta Obstet Gynecol Scand.

[12] Klebanoff SJ, Coombs RW (1991). Viricidal effect of Lactobacillus acidophilus on human immunodeficiency virus type 1: possible role in heterosexual transmission.. J Exp Med.

[13] Fontaine EA, Taylor-Robinson D (1990). Comparison of quantitative and qualitative methods of detecting hydrogen peroxide produced by human vaginal strains of lactobacilli.. J Appl Bacteriol.

[14] Tao L, Pavlova S, Anzinger J, Carlson S, Jacobs A (2005). Fighting HIV with its natural enemy: Mannose-specific capture of HIV by Lactobacillus.. http://wwwasmorg/ASM/files/ccLibraryFiles/Filename/000000001988/Beneficial%20Micrboes%20Program%20and%20Abstract%20Bookpdf.

[15] Reid G, Gibson GR, Rastall RA (2006). Extra intestinal effects of prebiotics and probiotics.. Prebiotics: Development and Applications.

[16] Buffa V, Stieh D, Mamhood N, Hu Q, Fletcher P (2009). Cyanovirin-N potently inhibits human immunodeficiency virus type 1 infection in cellular and cervical explant models.. J Gen Virol.

[17] O'Keefe BR, Vojdani F, Buffa V, Shattock RJ, Montefiori DC (2009). Scaleable manufacture of HIV-1 entry inhibitor griffithsin and validation of its safety and efficacy as a topical microbicide component.. Proc Natl Acad Sci U S A.

[18] Douek DC, Roederer M, Koup RA (2009). Emerging concepts in the immunopathogenesis of AIDS.. Annu Rev Med.

[19] Bolton M, van der Straten A, Cohen CR (2008). Probiotics: potential to prevent HIV and sexually transmitted infections in women.. Sex Transm Dis.

[20] Rabe LK, Hillier SL (2003). Optimization of media for detection of hydrogen peroxide production by Lactobacillus species.. J Clin Microbiol.

[21] Josephy PD, Eling T, Mason RP (1982). The horseradish peroxidase-catalyzed oxidation of 3,5,3′,5′-tetramethylbenzidine. Free radical and charge-transfer complex intermediates.. J Biol Chem.

[22] Al-Mushrif S, Jones BM (1998). A study of the prevalence of hydrogen peroxide generating Lactobacilli in bacterial vaginosis: the determination of H2O2 concentrations generated, in vitro, by isolated strains and the levels found in vaginal secretions of women with and without infection.. J Obstet Gynaecol.

[23] Song YL, Kato N, Matsumiya Y, Liu CX, Kato H (1999). Identification of and hydrogen peroxide production by fecal and vaginal lactobacilli isolated from Japanese women and newborn infants.. J Clin Microbiol.

[24] Schellenberg JJ, Links MG, Hill JE, Dumonceaux TJ, Kimani J (2011). Molecular definition of vaginal microbiota in East African commercial sex workers.. Appl Environ Microbiol.

[25] Hill JE, Penny SL, Crowell KG, Goh SH, Hemmingsen SM (2004). cpnDB: a chaperonin sequence database.. Genome Res.

[26] Hummelen R, Fernandes AD, Macklaim JM, Dickson RJ, Changalucha J (2010). Deep Sequencing of the Vaginal Microbiota of Women with HIV.. PLoS One.

[27] Schellenberg J, Blake Ball T, Lane M, Cheang M, Plummer F (2008). Flow cytometric quantification of bacteria in vaginal swab samples self-collected by adolescents attending a gynecology clinic.. J Microbiol Methods.

[28] Fowke KR, Dong T, Rowland-Jones SL, Oyugi J, Rutherford WJ (1998). HIV type 1 resistance in Kenyan sex workers is not associated with altered cellular susceptibility to HIV type 1 infection or enhanced beta-chemokine production.. AIDS Res Hum Retroviruses.

[29] Fowke KR, Nagelkerke NJ, Kimani J, Simonsen JN, Anzala AO (1996). Resistance to HIV-1 infection among persistently seronegative prostitutes in Nairobi, Kenya.. Lancet.

[30] Schellenberg J, Links MG, Hill JE, Dumonceaux TJ, Peters GA (2009). Pyrosequencing of the chaperonin-60 universal target as a tool for determining microbial community composition.. Appl Environ Microbiol.

[31] Kilic AO, Pavlova SI, Alpay S, Kilic SS, Tao L (2001). Comparative study of vaginal Lactobacillus phages isolated from women in the United States and Turkey: prevalence, morphology, host range, and DNA homology.. Clin Diagn Lab Immunol.

[32] Ison CA, Hay PE (2002). Validation of a simplified grading of Gram stained vaginal smears for use in genitourinary medicine clinics.. Sex Transm Infect.

[33] Nugent RP, Krohn MA, Hillier SL (1991). Reliability of diagnosing bacterial vaginosis is improved by a standardized method of gram stain interpretation.. J Clin Microbiol.

[34] Cardinale M, Brusetti L, Quatrini P, Borin S, Puglia AM (2004). Comparison of different primer sets for use in automated ribosomal intergenic spacer analysis of complex bacterial communities.. Appl Environ Microbiol.

[35] Saldanha AJ (2004). Java Treeview–extensible visualization of microarray data.. Bioinformatics.

[36] Hill JE, Paccagnella A, Law K, Melito PL, Woodward DL (2006). Identification of Campylobacter spp. and discrimination from Helicobacter and Arcobacter spp. by direct sequencing of PCR-amplified cpn60 sequences and comparison to cpnDB, a chaperonin reference sequence database.. J Med Microbiol.

[37] Pearson WR, Lipman DJ (1988). Improved tools for biological sequence comparison.. Proc Natl Acad Sci U S A.

