# Supplementary material for: Selection, Phenotyping and Identification of Acid and Hydrogen Peroxide Producing Bacteria from Vaginal Samples of Canadian and East African Women
Source: PLoS One. 2012 Jul 23;7(7):e41217. doi: 10.1371/journal.pone.0041217 (PMC3402533; doi:10.1371/journal.pone.0041217)
Supplement: Table S1 — Complete list and phylogenetic/phenotypic details of isolates. (PDF) [file pone.0041217.s001.pdf]

Table S1: Complete list and phylogenetic/phenotypic details of isolates

				ISR peaks				Phenotype		
Group	*Isolate*	*Seq'd*	*cpn60 UT Nearest Neighbour*	*Major*	*Minor1*	*Minor2*	*Minor3*	*Acid+*	*H_2O_2+*	*Double-strong*
L. sp. N6	M018-04	Y	*Lactobacillus* sp. N6 100%	284	290	488		0	1	
	M051-04	Y	*Lactobacillus* sp. N6 97%	368	290	251	488	1	0	
	M051-05	Y	*Lactobacillus* sp. N6 98%	290	252	488		0	0	
	M161-01	Y	*Lactobacillus* sp. N6 97%	289	313	488	415	2	2	
	M170-03	Y	*Lactobacillus* sp. N6 96%	290	488			0	1	
	M207-02	Y	*Lactobacillus* sp. N6 100%	290	488			0	1	
	P001-01	Y	*Lactobacillus* sp. N6 97%	289	490			0	0	
	P009-08	Y	*Lactobacillus* sp. N6 97%	293	304	315		0	1	
	P011-07	Y	*Lactobacillus* sp. N6 96%	290	488			3	3	Y
	M001-03			290	488			0	1	
	M001-04			290	488			0	1	
	M001-05			290	488			0	1	
	M001-06			290	488			0	1	
	M007-01			290	488			0	0	
	M007-02			290	488			0	0	
	M007-03			290	488	670		0	0	
	M007-04			290	488			0	0	
	M007-05			290	488			0	0	
	M007-06			290	488			0	0	
	M018-03			290	488			0	1	
	M018-06			290	488			0	1	
	M022-03			290	488			0	1	
	M024-01			290				0	0	
	M024-03			290	489			0	0	
	M024-04			290	489			0	0	
	M024-05			290	489			0	0	
	M025-03			290	488			0	0	
	M025-04			290	488			0	0	
	M025-06			290	488			0	1	
	M027-02			290	488			1	1	
	M038-02			290	488			0	0	
	M038-03			290	488			0	0	
	M050-01			290	489			1	1	
	M050-04			290	488			0	0	
	M051-01			290	488			0	0	
	M051-02			290	488	251		0	0	
	M051-06			290	252	488		0	1	
	M061-05			290	489			0	1	
	M067-02			290	489	383		1	2	
	M072-01			290	489			0	1	

Table S1: Complete list and phylogenetic/phenotypic details of isolates

Group	Isolate	Seq'd	cpn60 UT Nearest Neighbour	ISR peaks				Phenotype		
				Major	Minor1	Minor2	Minor3	Acid+	H_2O_2+	Double-strong
L. sp. N6 (cont.)	M072-02			290	489			0	1	
	M080-03			290	488			0	1	
	M080-04			290	489			0	1	
	M087-01			290	489	271		0	1	
	M101-02			290	489	251		0	1	
	M101-03			290	489	251		0	1	
	M103-03			290	489			0	1	
	M103-04			290	489			0	1	
	M103-05			290	489			0	1	
	M110-03			290	488			0	1	
	M113-01			290	489			0	0	
	M113-02			290	489	276		0	0	
	M113-03			290	489			0	0	
	M113-04			290	489			0	0	
	M122-04			290	488			0	1	
	M124-02			289	490			0	0	
	M124-03			289	490	274		0	0	
	M126-04			290	489			0	1	
	M126-05			290	489	260		0	1	
	M126-07			290	489			1	1	
	M127-04			290	489			0	1	
	M131-01			290	489			1	0	
	M131-03			290	489			1	0	
	M135-07			290	489			1	2	
	M135-09			290	489			1	2	
	M139-01			290	489			0	1	
	M139-02			290	489			0	1	
	M139-03			290	489			0	1	
	M139-05			290	489			0	1	
	M145-01			290	488			0	1	
	M145-03			290	488			0	1	
	M145-04			290	488			0	0	
	M145-06			290	488			0	0	
	M155-01			289	489			0	0	
	M155-02			289	490			0	0	
	M155-05			289	490			0	0	
	M157-01			290	488			0	0	
	M157-02			290	488			0	1	
	M161-03			290	488			2	2	
	M161-05			289	488			0	0	

Table S1: Complete list and phylogenetic/phenotypic details of isolates

Group	Isolate	Seq'd	cpn60 UT Nearest Neighbour	ISR peaks				Phenotype		
				Major	*Minor1*	*Minor2*	*Minor3*	*Acid+*	*H_2O_2+*	*Double-strong*
L. sp. N6 (cont.)	M161-06			289	489			0	1	
	M162-02			290	488			0	1	
	M162-05			290	488			0	1	
	M163-01			290	488			0	1	
	M163-02			290	489			0	1	
	M165-02			289	488			0	1	
	M165-03			289	488			0	0	
	M165-04			289	488			0	1	
	M167-01			289	487			1	1	
	M170-04			290	489			0	1	
	M178-02			290	488			0	1	
	M200-01			290	488	252		0	0	
	M200-04			290	488			0	0	
	M203-01			289	490			0	0	
	M203-02			289	490			0	0	
	M203-03			289	490			0	0	
	M205-02			290	488			0	1	
	M205-04			290	488			0	1	
	M205-07			290	488	353		1	0	
	P001-06			289	490			0	0	
	P001-10			289	490			0	0	
	P001-17			289	490			0	0	
	P001-18			289	490			0	0	
	P009-04			289	490			0	1	
	P009-05			289	490			0	1	
	P009-06			289	490			0	1	
	P009-07			289	490	274		0	1	
	P011-08			290	488			3	3	Y
L. sp. N27	M104-08	Y	*Lactobacillus* sp. N6 83%	283	480			2	1	
	M012-01			283	480			2	1	
	M012-02			283	290	480		2	1	
	M012-03			283	480			2	1	
	M012-04			283	480	112		2	1	
	M013-01			283	479			2	2	
	M013-04			283	479			2	2	
	M061-02			283	145	480		1	1	
	M061-03			283	480			1	1	
	M061-04			283				1	1	
	M104-02			283	480			2	0	
	M131-02			283	479			1	0	

Table S1: Complete list and phylogenetic/phenotypic details of isolates

Group	Isolate	Seq'd	cpn60 UT Nearest Neighbour	ISR peaks				Phenotype		
				Major	*Minor1*	*Minor2*	*Minor3*	*Acid+*	*H_2O_2+*	*Double-strong*
L. sp. N27 (cont.)	M135-06			283	479			2	2	
	M135-08			283	479			2	2	
	M136-03			283	480			2	1	
	M156-01			283	130	480		2	2	
	M156-02			283	130	145	480	2	2	
	P009-01			283	479			3	0	
	P009-09			283	479			3	0	
	P009-10			283	479			3	0	
	P022-01			283	479			2	0	
	P022-03			283	479			2	0	
	P030-08			283	479			2	0	
	P030-14			283	479			2	0	
L. oris	P025-05	Y	*Lactobacillus oris* F0423 100%	291	183	486		2	2	
L. sp. N19	P025-07	Y	Lactobacillus reuteri ATCC23272 93%	290	485			2	2	
	P025-08	Y	Lactobacillus reuteri ATCC23272 93%	290	485			2	2	
L. reuteri	M091-02	Y	*Lactobacillus reuteri* ATCC23272 100%	289	491			2	2	
	M091-03			289	491			2	2	
	M091-07			289	491			3	3	Y
	M135-03			289	491			0	1	
	M204-02			289	491			1	2	
	M204-03			289	491			1	2	
	M204-04			289	491			1	2	
	M204-05			289	491			1	2	
	M204-06			289	491			1	2	
	P002-05			289				0	0	
	P002-18			289				0	1	
L. coleohominis	M050-05	Y	Lactobacillus coleohominis 101-4-CHN 98%	230	284	489		0	1	
	M080-05	Y	Lactobacillus coleohominis 101-4-CHN 100%	146	284	489		0	1	
	M212-01	Y	Lactobacillus coleohominis 101-4-CHN 100%	284	488			0	1	
	M013-03			284	488			1	1	
	M013-06			284	489			1	1	
	M018-02			284	488			0	1	
	M018-05			284	488			0	1	
	M085-05			284	489			1	1	
	M085-08			284	489			1	1	
	M110-02			284	489			0	1	
	M110-05			284	489			0	1	
	M127-05			284	489			0	1	
	M177-01			284	489			0	1	
	M177-02			284	489			0	1	

Table S1: Complete list and phylogenetic/phenotypic details of isolates

Group	Isolate	Seq'd	cpn60 UT Nearest Neighbour	ISR peaks				Phenotype		
				Major	*Minor1*	*Minor2*	*Minor3*	*Acid+*	*H_2O_2+*	*Double-strong*
L. coleohominis (cont.)	M177-03			284	489			0	1	
	M177-04			284	489	270		0	1	
	M177-05			284	488			0	1	
	M177-06			284	488			0	1	
	M178-03			284	488			0	1	
	M178-05			284	488			0	1	
	M178-08			284	488			0	1	
	M190-01			284	489			0	1	
	M190-02			284	488			0	1	
	M200-02			284	489			0	1	
	M200-03			284	290	488		0	1	
	M200-05			284	489			0	1	
	M200-07			284	488			0	1	
	M215-01			284	489			1	2	
	M215-07			284	488			2	1	
	M225-02			284	489			0	1	
P. pentosaceus	M221-02	Y	*Pediococcus pentosaceus* ATCC33316 99%	315	505			3	2	Y
	M221-03			315	505			3	2	Y
	M221-05			315	505	263		3	2	Y
W. cibaria	M166-01	Y	*Weissella cibaria* KACC11862 100%	337	356	534	460	2	2	
L. plantarum	M085-07	Y	*Lactobacillus plantarum* ATCC14431 99%	291	521	272		3	1	
L. ruminis	M067-01	Y	*Lactobacillus ruminis* ATCC2564 99%	300	400	522	500	3	3	Y
	M094-01	Y	*Lactobacillus ruminis* ATCC2564 99%	300	400	522	500	3	2	Y
	M109-03	Y	*Lactobacillus ruminis* ATCC2564 99%	299	401	500	522	2	2	
	M135-05	Y	*Lactobacillus ruminis* ATCC2564 99%	300	401	500	522	2	2	
	M094-02			299	400	522	500	3	2	Y
	M109-04			300	401	500	522	2	2	
L. animalis	M023-03	Y	*Lactobacillus animalis* ATCC35046 98%	288	386			3	3	Y
	M027-03	Y	*Lactobacillus animalis* ATCC35046 98%	289	386	471	484	3	3	Y
	M085-04	Y	*Lactobacillus animalis* ATCC35046 98%	288	386	484	471	2	1	
	M085-09	Y	*Lactobacillus animalis* ATCC35046 98%	288	386	471	484	3	3	Y
	M212-02	Y	*Lactobacillus animalis* ATCC35046 98%	289	386	484	471	3	2	Y
	M054-01			289	386	484	471	3	0	
	M054-05			289	386	484	471	3	0	
	M054-06			289	386	484	471	3	0	
	M057-01			289	386	484	471	2	1	
	M057-02			289	386	484	471	2	1	
	M059-01			288	386	484	471	2	1	
	M077-01			288	386	484	471	3	0	
	M079-01			288	386	471	484	3	0	

Table S1: Complete list and phylogenetic/phenotypic details of isolates

				ISR peaks				Phenotype		
Group	*Isolate*	*Seq'd*	*cpn60 UT Nearest Neighbour*	*Major*	*Minor1*	*Minor2*	*Minor3*	*Acid+*	*H_2O_2+*	*Double-strong*
L. animalis (cont.)	M079-02			288				3	0	
	M079-05			288	386	471	484	3	0	
	M083-01			288	386	484	471	3	0	
	M083-03			288	386	484	471	3	0	
	M085-02			288	386	484	471	3	0	
	M094-05			288	386	484	471	3	0	
	M212-07			289	386	484	471	3	2	Y
	M225-03			288	386	484	471	2	2	
	M225-05			288	386	484	471	3	0	
	M225-08			288	386	471	484	3	0	
	M225-10			288	386	471	484	3	0	
L. salivarius	M013-02	Y	*Lactobacillus salivarius* ATCC11741 100%	293				3	0	
	M016-02	Y	*Lactobacillus salivarius* ATCC11741 100%	293				3	3	Y
	M037-01	Y	*Lactobacillus salivarius* ATCC11741 100%	293				3	3	Y
	M085-03	Y	*Lactobacillus salivarius* ATCC11741 97%	293	509			3	3	Y
	M104-05	Y	*Lactobacillus salivarius* ATCC11741 100%	293	509			3	3	Y
	M136-01	Y	*Lactobacillus salivarius* ATCC11741 100%	293	509			3	3	Y
	M161-04	Y	*Lactobacillus salivarius* ATCC11741 100%	293	509			3	3	Y
	M212-03	Y	*Lactobacillus salivarius* ATCC11741 97%	293	509			2	1	
	M212-08	Y	*Lactobacillus salivarius* ATCC11741 100%	293	509			3	1	
	M216-01	Y	*Lactobacillus salivarius* ATCC11741 97%	293	509			3	1	
	M225-04	Y	*Lactobacillus salivarius* ATCC11741 100%	293	509			3	3	Y
	M054-03			293	509			2	1	
	M054-04			293	509	279		2	1	
	M054-07			293	509			1	1	
	M059-02			293	509	279		2	2	
	M068-01			293	509			3	0	
	M068-02			293	509			3	0	
	M077-02			293	509			1	1	
	M077-03			293	509			1	1	
	M104-01			293	509			3	3	Y
	M104-06			293	509	278		3	3	Y
	M136-02			293	509			3	3	Y
	M212-04			293	509			1	1	
	M216-02			293	509			3	1	
	M216-03			293	509			3	1	
	M216-04			293	509			3	0	
	P025-01			293	510			3	0	
	P025-02			293	509	276		3	1	
	P025-03			293	510			3	1	

Table S1: Complete list and phylogenetic/phenotypic details of isolates

				**ISR peaks**				**Phenotype**		
Group	*Isolate*	*Seq'd*	*cpn60 UT Nearest Neighbour*	*Major*	*Minor1*	*Minor2*	*Minor3*	*Acid+*	*H_2O_2+*	*Double-strong*
L. salivarius (cont.)	P025-04			293	510	279		3	0	
L. delbrueckii	M091-01	Y	*Lactobacillus delbrueckii* ATCC12315 99%	304	545			2	2	
	M091-08	Y	*Lactobacillus delbrueckii* ATCC12315 99%	304	545			3	3	Y
	M091-05			304	545			3	3	Y
L. gasseri	M018-01	Y	*Lactobacillus gasseri* ATCC33323 100%	308	538			1	1	
	M054-02	Y	*Lactobacillus gasseri* ATCC33323 99%	289	308	386	471	3	3	Y
	M091-06	Y	*Lactobacillus gasseri* ATCC33323 97%	308	538			1	1	
	M091-10	Y	*Lactobacillus gasseri* ATCC33323 97%	308	538	287	294	1	1	
	M104-07	Y	*Lactobacillus gasseri* ATCC33323 97%	308	538			1	1	
	M127-06	Y	*Lactobacillus gasseri* ATCC33323 100%	308	538			1	1	
	M136-04	Y	*Lactobacillus gasseri* ATCC33323 97%	308	538			1	1	
	M170-06	Y	*Lactobacillus gasseri* ATCC33323 97%	308	538			1	1	
	P005-02	Y	*Lactobacillus gasseri* ATCC33323 100%	308	538			1	1	
	P011-01	Y	*Lactobacillus gasseri* ATCC33323 100%	306	538			1	1	
	M016-01			308	538			2	1	
	M016-05			308	538			2	1	
	M085-06			308	538			1	1	
	M091-04			308	538			1	1	
	M104-03			308	538			1	1	
	M104-04			308	538	294		1	1	
	M110-01			308	538			0	1	
	M110-04			308	538			0	1	
	M127-02			308	538			1	1	
	M127-03			308	538			1	1	
	M135-04			308	539			1	0	
	M155-03			308	538			1	1	
	M155-04			308	289	538	490	1	1	
	M155-06			308	538			1	1	
	M161-02			308	539			1	1	
	M166-02			308	538			1	1	
	M170-01			308	538			1	1	
	M170-10			308	538			1	1	
	M215-02			308	538			1	2	
	P005-03			308	538			1	1	
	P005-04			308	538			1	1	
	P005-05			308	538			1	1	
	P005-06			308	538			1	1	
	P005-07			308	538			1	1	
	P005-08			308	538			1	1	
	P005-09			308	538			1	1	

Table S1: Complete list and phylogenetic/phenotypic details of isolates

				ISR peaks				Phenotype		
Group	*Isolate*	*Seq'd*	*cpn60 UT Nearest Neighbour*	*Major*	*Minor1*	*Minor2*	*Minor3*	*Acid+*	*H_2O_2+*	*Double-strong*
L. gasseri (cont.)	P005-10			308	538			1	1	
	P005-11			308	538			1	1	
	P005-12			308	538			1	1	
	P009-03			308	539			1	1	
	P011-02			306	539			1	1	
	P011-03			306	539	292		1	1	
	P011-09			306	539			1	1	
	P011-10			306	539			1	1	
	P021-01			308				1	0	
	P021-03			308				1	0	
	P021-04			308				1	0	
	P021-05			308				1	0	
	P021-06			308				1	0	
	P021-07			308	294			1	0	
	P021-08			308				1	0	
	P021-09			308				1	0	
	P021-12			308				1	0	
	P021-13			308				1	0	
	P021-14			308	294			1	0	
	P021-15			308				1	0	
	P022-04			306	539			1	1	
	P022-05			306	539			1	1	
	P022-06			306	539			1	1	
L. jensenii	M124-01	Y	*Lactobacillus jensenii* ATCC25258 100%	288	525			0	1	
	M124-07	Y	*Lactobacillus jensenii* ATCC25258 100%	288	525	268	273	0	1	
	M178-04	Y	*Lactobacillus jensenii* ATCC25258 95%	288	112	526		1	0	
	M207-01	Y	*Lactobacillus jensenii* ATCC25258 100%	288	525			0	1	
	P013-08	Y	*Lactobacillus jensenii* ATCC25258 95%	288	112	526		0	1	
	P014-07	Y	*Lactobacillus jensenii* ATCC25258 95%	288	526	268		1	2	
	P014-09	Y	*Lactobacillus jensenii* ATCC25258 95%	526	288	268	274	1	2	
	M038-01			288	526			1	1	
	M038-04			288	526			1	1	
	M038-06			288	526	273		2	1	
	M050-02			288	112	526		0	0	
	M050-03			112	274	288	526	0	0	
	M050-06			288	526	112		1	1	
	M067-03			288	526			1	2	
	M080-01			288	525			0	0	
	M080-02			288	525			0	1	
	M080-06			288	525			0	1	

Table S1: Complete list and phylogenetic/phenotypic details of isolates

Group	Isolate	Seq'd	cpn60 UT Nearest Neighbour	ISR peaks				Phenotype		
				Major	*Minor1*	*Minor2*	*Minor3*	*Acid+*	*H_2O_2+*	*Double-strong*
L. jensenii (cont.)	M101-01			288	525			0	0	
	M101-04			288	525			0	0	
	M101-06			288	525			0	0	
	M101-07			288	525	273		0	0	
	M122-01			288				0	1	
	M122-02			288				0	1	
	M122-03			288	112			0	1	
	M122-05			288	112	526		0	1	
	M122-06			288	112	526		0	1	
	M124-04			288	525			0	1	
	M124-05			288	525			0	1	
	M178-01			288	112	526		1	0	
	M178-06			288				1	1	
	M183-01			288	525			0	0	
	M183-06			288	525			1	0	
	M183-07			288	525			1	0	
	M183-08			288	525			1	0	
	M207-03			288	525			0	1	
	P009-02			288	525	274		0	1	
	P013-03			288	112	526		0	1	
	P013-04			288	112	526		0	1	
	P013-11			288	112	526		0	1	
	P014-01			288	526	269		1	2	
	P014-02			288	526	269		1	2	
	P014-03			288	526			1	2	
	P014-04			288	526	269		1	2	
	P014-05			288	526	269		1	2	
	P014-06			288	526	269		1	2	
	P014-08			288	526	268		1	2	
	P014-10			288	526			1	2	
	P014-11			288	526			1	2	
	P014-12			288	526			1	2	
	P016-01			288	525			1	1	
	P016-02			288	525			1	1	
	P016-03			288	525			1	1	
	P016-04			288	526			1	1	
	P016-05			288	525			1	1	
	P016-07			288	526			1	1	
	P016-08			288	526	273		1	1	
	P016-09			288	525	274		1	1	

Table S1: Complete list and phylogenetic/phenotypic details of isolates

Group	Isolate	Seq'd	cpn60 UT Nearest Neighbour	ISR peaks				Phenotype		
				Major	Minor1	Minor2	Minor3	Acid+	H_2O_2+	Double-strong
L. jensenii (cont.)	P016-10			288	525			1	1	
	P016-11			288	525			1	1	
	P016-12			288				1	1	
	P025-06			288	526			1	1	
	P025-10			288	526			0	1	
L. crispatus	M001-01	Y	*Lactobacillus crispatus* CUTC3695 100%	293	546			0	1	
	M024-02	Y	*Lactobacillus crispatus* CUTC3695 100%	293				0	0	
	M082-01	Y	*Lactobacillus crispatus* CUTC3695 100%	162	294			1	1	
	M082-04	Y	*Lactobacillus crispatus* CUTC3695 100%	294				1	1	
	P001-02	Y	*Lactobacillus crispatus* CUTC3695 100%	293	162			0	0	
	P001-09	Y	*Lactobacillus crispatus* CUTC3695 100%	293	546			0	0	
	P001-12	Y	*Lactobacillus crispatus* CUTC3695 100%	293	162			0	0	
	P001-15	Y	*Lactobacillus crispatus* CUTC3695 100%	293				0	0	
	P002-07	Y	*Lactobacillus crispatus* CUTC3695 100%	293	99	124		0	0	
	P002-10	Y	*Lactobacillus crispatus* CUTC3695 100%	293	162	309		0	0	
	P002-16	Y	*Lactobacillus crispatus* CUTC3695 100%	99				0	0	
	M001-02			293	546			0	1	
	M003-01			293	547			1	1	
	M003-02			293	547			1	1	
	M003-03			293				1	1	
	M006-01			293	162	547	279	1	1	
	M006-02			293	162	547		1	1	
	M006-03			293	162	547		1	1	
	M066-01			293	547			0	1	
	M066-02			293	547			0	1	
	M066-03			293	547			0	0	
	M066-04			293	547	162		0	0	
	M076-01			294	547			1	2	
	M076-02			294	547			1	2	
	M076-04			294	547			1	2	
	M076-05			294	547			1	2	
	M081-01			293	546			0	1	
	M082-03			293	547			1	1	
	M082-05			294				1	1	
	M082-06			294				0	1	
	M085-01			293	546	127		1	2	
	M103-02			294	547			0	1	
	M126-01			294	547			0	1	
	M126-02			294	547			0	1	
	M127-01			293	546			1	1	

Table S1: Complete list and phylogenetic/phenotypic details of isolates

Group	Isolate	Seq'd	cpn60 UT Nearest Neighbour	ISR peaks				Phenotype		
				Major	Minor1	Minor2	Minor3	Acid+	H_2O_2+	Double-strong
L. crispatus (cont.)	M131-04			294	547	156	309	1	0	
	M132-01			294	547			0	1	
	M132-02			294	547	309	156	0	0	
	M132-03			294	547			1	0	
	M145-02			293	546			0	0	
	M162-01			293	546			1	1	
	M162-03			293	162			1	1	
	M183-02			293	546			0	0	
	M183-03			293	546			0	0	
	M183-04			293	546			0	0	
	M183-05			293	546			0	0	
	M199-01			293	546			0	0	
	M204-01			293	546			1	1	
	M205-01			293	546			0	0	
	M205-03			293	546			0	0	
	M205-05			293	546			0	0	
	M205-06			293	546			0	0	
	M220-01			293	546			0	0	
	M220-02			293	546			0	0	
	P001-03			293	162	309	99	0	0	
	P001-04			293	162	154		0	0	
	P001-05			293				0	0	
	P001-07			293				0	0	
	P001-08			293				0	0	
	P001-11			293	162	309	99	0	0	
	P001-13			293	162	99	309	0	1	
	P001-14			293	162			0	1	
	P001-16			293				0	0	
	P002-01			293	162			0	0	
	P002-02			293	162	99	309	0	0	
	P002-03			293	162	309		0	0	
	P002-04			293	162	309	99	0	0	
	P002-06			293	99			0	0	
	P002-08			293	162			0	0	
	P002-09			293				0	0	
	P002-11			293				0	0	
	P002-12			293	546			0	0	
	P002-14			293	162			0	0	
	P002-15			293	162	309	99	0	0	
	P002-17			293				0	0	

Table S1: Complete list and phylogenetic/phenotypic details of isolates

Group	Isolate	Seq'd	cpn60 UT Nearest Neighbour	ISR peaks				Phenotype		
				Major	*Minor1*	*Minor2*	*Minor3*	*Acid+*	*H_2O_2+*	*Double-strong*
L. crispatus (cont.)	P011-04			293	546			1	2	
	P011-05			293	546			1	2	
	P011-06			293	546	162		1	2	
	P011-11			293	546	162	279	1	2	
	P011-12			293	546	273	162	1	2	
	P013-01			293	162			1	2	
	P013-02			293				1	2	
	P013-05			293	546			1	2	
	P013-06			293	162			1	2	
	P013-07			293				1	2	
	P013-09			293	546			1	2	
	P013-10			293				1	2	
	P013-12			293	546			1	2	
	P021-02			293	546			1	1	
	P021-10			293	546			1	1	
	P021-11			293	546			1	1	
	P021-16			293	546			1	1	
	P021-17			293	546			1	1	
	P021-18			293	546			1	1	
	P022-02			293	546			0	1	
	P030-07			293	194	162	546	1	1	
	P031-01			293				0	1	
	P031-02			293	547			0	1	
	P031-03			293	162	547	279	0	1	
	P031-04			293	547	162		0	1	
	P031-05			293	162	547		0	1	
	P031-06			293	547	162		0	1	
	P031-07			293	547			0	1	
	P031-08			293	547			0	1	
	P031-09			293	547			0	1	
	P031-10			293	547			0	1	
	P031-11			293	162	547	279	0	1	
	P031-12			293	547	162	279	0	1	
En. faecalis	M161-07	Y	*Enterococcus faecalis* ATCC49332 99%	313	414			1	0	
	P030-11	Y	*Enterococcus faecalis* ATCC49332 99%	313	415			2	0	
	M006-06			313	415			2	2	
	M013-05			313	415			2	1	
	M037-05			415	189	171		2	1	
	M081-03			313	415	293		2	1	
	M145-05			313	415			2	1	

Table S1: Complete list and phylogenetic/phenotypic details of isolates

Group	Isolate	Seq'd	cpn60 UT Nearest Neighbour	ISR peaks				Phenotype		
				Major	Minor1	Minor2	Minor3	Acid+	H_2O_2+	Double-strong
En. faecalis (cont.)	M163-03			313	415			1	0	
En. raffinosus	M083-02	Y	*Enterococcus raffinosus* ATCC49427 100%	311	413			2	3	Y
	M083-04	Y	*Enterococcus raffinosus* ATCC49427 100%	311	413			2	3	Y
S. bovis	M023-01	Y	*Streptococcus bovis* ATCC9809 99%	361				3	2	Y
	M023-06	Y	*Streptococcus bovis* ATCC9809 99%	361				2	2	
	M029-04	Y	*Streptococcus bovis* ATCC9809 99%	361	148	305		2	1	
	M023-02			361				3	2	Y
	M023-05			361				3	2	Y
	M025-01			361				2	2	
	M025-02			361				2	2	
	M025-05			361				2	2	
	M027-01			361				2	2	
	M027-04			361				1	2	
	M029-01			361				2	1	
	M029-02			361				2	1	
	M029-03			361				2	1	
	M029-05			361				2	1	
	M079-03			361				2	1	
	M079-04			361				2	1	
	M081-02			361				3	2	Y
S. lutetiensis	M086-01	Y	*Streptococus lutetiensis* BAA-103 100%	360				3	1	
	M094-09	Y	*Streptococus lutetiensis* BAA-103 100%	360				1	0	
	M103-01	Y	*Streptococus lutetiensis* BAA-103 100%	360				3	3	Y
	P005-01	Y	*Streptococus lutetiensis* BAA-103 100%	360				2	0	
	M067-06			360	383			1	2	
	M079-06			360				1	1	
	M086-02			360				3	0	
	M086-03			360				3	0	
	M086-04			360				3	0	
	M094-04			360				3	1	
	M103-08			360	342			1	0	
	M156-03			360				2	2	
	M156-04			360				1	2	
	M156-05			360				1	2	
	M157-03			360				2	0	
	P030-01			360				2	0	
	P030-02			360				2	0	
	P030-16			360				1	0	
	P030-17			360				1	0	
	P030-18			360				1	0	

Table S1: Complete list and phylogenetic/phenotypic details of isolates

Group	Isolate	Seq'd	cpn60 UT Nearest Neighbour	ISR peaks				Phenotype		
				Major	Minor1	Minor2	Minor3	Acid+	H_2O_2+	Double-strong
S. agalactiae	M016-06	Y	*Streptococcus agalactiae* ATCC12386 100%	368				2	1	
	M038-05	Y	*Streptococcus agalactiae* ATCC12386 100%	368				2	0	
	M059-03	Y	*Streptococcus agalactiae* ATCC12386 100%	368				2	2	
	M059-05	Y	*Streptococcus agalactiae* ATCC12386 100%	367				2	2	
	M027-06			368				1	2	
	M059-06			368				2	2	
	M067-05			368				2	2	
	M068-03			368				2	2	
	M068-05			367				2	2	
	M097-01			368				2	2	
	M097-02			367				2	2	
	M097-03			368				2	2	
	M097-04			368				2	2	
	M099-03			368				2	2	
	M099-04			368				2	2	
	M099-05			367				2	2	
	M139-04			367				2	2	
	M139-08			368				2	2	
	M148-03			367				2	2	
	M148-04			368				2	2	
	M148-05			368				2	2	
	M156-06			368				1	1	
	M167-03			367				2	0	
	M167-04			368				2	0	
	M175-02			368				2	2	
	M197-02			368				2	2	
	M215-03			367				2	2	
	M215-04			368	354			2	1	
	M220-03			368				2	2	
	M220-05			368				2	1	
	M220-06			368				2	2	
S. sp. N80	M068-04	Y	*Streptococcus mitis* ATCC49456 94%	331				1	2	
	M081-05	Y	*Streptococcus mitis* ATCC49456 94%	331				1	1	
	M022-04			331				1	2	
	M023-04			331				1	2	
	M067-04			331				1	2	
	M081-04			331				1	1	
	M081-06			331				1	1	
	M087-02			331				1	2	
	M190-03			331				1	1	

Table S1: Complete list and phylogenetic/phenotypic details of isolates

Group	Isolate	Seq'd	cpn60 UT Nearest Neighbour	ISR peaks				Phenotype		
				Major	Minor1	Minor2	Minor3	Acid+	H_2O_2+	Double-strong
S. sp. N80 (cont.)	M190-06			331				1	1	
	M190-07			331				1	1	
	M197-01			331				1	1	
	M197-04			331				1	1	
	M212-05			331				1	1	
	M212-09			331				1	2	
	M225-06			331				1	1	
S. sp. N127	M005-04	Y	*Streptococcus pneumoniae* SP14-BS69 95%	335				1	1	
	M016-03	Y	*Streptococcus pneumoniae* SP14-BS69 95%	334				1	1	
	M162-04	Y	*Streptococcus pneumoniae* SP14-BS69 95%	335				1	1	
	M005-05			311	335	337		0	0	
	M016-04			334				1	1	
	M037-06			335				1	1	
	M052-04			335	144			1	1	
	M109-05			336				1	2	
	M109-06			335				1	2	
	M161-10			335				1	2	
	P030-03			335	360	460	368	2	1	
S. anginosus	M003-04	Y	*Streptococcus anginosus* ATCC33397 96%	479				2	1	
	M037-03	Y	*Streptococcus anginosus* ATCC33397 95%	477	590			2	2	
	M037-04	Y	*Streptococcus anginosus* ATCC33397 95%	477	590			2	2	
	M003-05			479				2	1	
	M037-02			477				2	2	
S. constellatus	M005-02	Y	*Streptococcus constellatus* ATCC27823 97%	383	224	325	441	1	1	
	M005-06	Y	*Streptococcus constellatus* ATCC27823 97%	383				1	1	
	M132-05	Y	*Streptococcus constellatus* ATCC27823 97%	382				0	0	
	M199-03	Y	*Streptococcus constellatus* ATCC27823 97%	383				3	0	
	M006-05			383				2	2	
	M022-01			383				1	2	
	M022-05			383				2	2	
	M031-04			383	459	235		2	2	
	M052-02			383				1	1	
	M052-03			383	369			1	1	
	M094-06			383				1	2	
	M099-01			383				2	2	
	M099-02			383				2	2	
	M100-03			383				2	2	
	M135-02			382				1	2	
	M166-03			383				1	2	
	M170-05			382				1	2	

Table S1: Complete list and phylogenetic/phenotypic details of isolates

Group	Isolate	Seq'd	cpn60 UT Nearest Neighbour	ISR peaks				Phenotype		
				Major	*Minor1*	*Minor2*	*Minor3*	*Acid+*	*H_2O_2+*	*Double-strong*
S. constellatus (cont.)	M181-01			383				2	2	
	M181-02			383				2	2	
	M199-04			382				1	1	
	M207-04			383				1	2	
	M207-06			383				2	2	
	M215-05			382				2	0	
	M215-08			382				2	0	
	M225-07			383				2	0	
	P009-11			383				1	1	
	P018-03			383				2	2	
	P018-04			383				2	2	
	P018-06			383				3	0	
	P025-11			383				1	2	
	P030-04			383				2	0	
	P030-15			383				2	1	
Pe. harei	M212-06	Y	*Peptoniphilus harei* ACS-146-V-Sch2b 98%	470				1	1	
Ba. cereus	M181-03	Y	*Bacillus cereus* NRRL569 98%	230	459			1	1	
Ge. haemolysans	M094-07	Y	*Gemella haemolysans* z1643 98%	301	508			1	2	
	M094-10	Y	*Gemella haemolysans* z1643 87%	298	504			1	2	
St. simulans	M132-04	Y	*Staphylococcus simulans* ATCC27848 99%	333	424	509		0	1	
St. haemolyticus	M006-04	Y	*Staphylococcus haemolyticus* ATCC29970 99%	532	554	647	206	1	0	
	M103-09	Y	*Staphylococcus haemolyticus* ATCC29970 100%	425	448	531	543	1	0	
St. hominis	M124-06	Y	*Staphylococcus hominis* ATCC27844 100%	388	409	503		1	0	
St. epidermidis	M167-02	Y	*Staphylococcus epidermidis* ATCC14990 98%	350	444	542		1	0	
	M167-05	Y	*Staphylococcus epidermidis* ATCC14990 98%	350	444	542		1	0	
	P006-01	Y	*Staphylococcus epidermidis* ATCC14990 100%	353	446			1	0	
	M003-06			353	445	187	542	1	0	
	M005-03			351	353	444	542	1	0	
	M087-04			351	444	542		1	1	
	M087-05			350	444	331	542	1	0	
	M110-06			350	459	583	444	1	0	
	M162-06			350	444	542		1	0	
	P006-04			353	446			1	0	
	P006-05			353	446			1	0	
	P006-06			353	446	542		1	0	
St. lugdunensis	M005-01	Y	*Staphylococcus lugdunensis* ATCC43809 99%	351	383	580	584	1	1	
	M024-06	Y	*Staphylococcus lugdunensis* ATCC43809 95%	351	583	446	607	1	0	
	M187-03	Y	*Staphylococcus lugdunensis* ATCC43809 100%	583	607	654	685	1	0	
St. piscifermentans	M197-03	Y	*Staphylococcus piscifermentans* ATCC51136 100%					2	0	
Ba. fragilis	M057-03	Y	*Bacteroides fragilis* ATCC25285 100%	575	581	100	168	1	0	

Table S1: Complete list and phylogenetic/phenotypic details of isolates

				ISR peaks				Phenotype		
Group	*Isolate*	*Seq'd*	*cpn60 UT Nearest Neighbour*	*Major*	*Minor1*	*Minor2*	*Minor3*	*Acid+*	*H_2O_2+*	*Double-strong*
Ba. fragilis (cont.)	M083-05	Y	*Bacteroides fragilis* ATCC25285 99%	570	575	579	168	1	0	
Pr. disiens	M027-05	Y	*Prevotella disiens* ATCC29426 99%	583	353	607		1	0	
Pr. bivia	M170-07	Y	*Prevotella bivia* ATCC29303 99%	537	771	522	791	1	0	
	M175-04	Y	*Prevotella bivia* ATCC29303 99%	543	774	529		1	0	
K. pneumoniae	M161-09	Y	*Klebsiella pneumoniae* ATCC13883 99%	291	435	427	284	1	0	
E. coli	M087-03	Y	*Escherichia coli* ATCC35401 99%	439	534	524		1	0	
	M126-03	Y	*Escherichia coli* ATCC35401 99%	439	534	429		0	1	
	M135-01	Y	*Escherichia coli* ATCC35401 99%	439	534	429		2	2	
	M045-06			439	429	524	534	1	0	
	M094-08			439	534	526		1	0	
N159	M157-04	Y	*Actinomyces* <80%	397	145	383	259	1	0	
Bif. longum	M022-02	Y	*Bifidobacterium longum* ATCC15707 100%	182	178	252		3	0	
	M045-01	Y	*Bifidobacterium longum* ATCC15707 100%	178	181	352	183	2	0	
	M051-03	Y	*Bifidobacterium longum* ATCC15707 100%	182	110	178	252	3	1	
	M052-01	Y	*Bifidobacterium longum* ATCC15707 95%	177	182	162	389	2	0	
	M109-01	Y	*Bifidobacterium longum* ATCC15707 98%	178	182	162		2	0	
	M158-01	Y	*Bifidobacterium longum* ATCC15707 100%	178	162			2	0	
	M199-02	Y	*Bifidobacterium longum* ATCC15707 99%	509	110	181	494	3	0	
	M094-03			510	193	181	496	3	0	
	P025-09			182	178	252	162	2	2	
	P025-12			177	182			2	0	
	P030-09			510	177	495		2	0	
	P030-10			178	162	510		2	0	
	P030-12			177	162			2	0	
	P030-13			177	182	162	171	2	0	
B. catenulatum	M052-06	Y	*Bifidobacterium catenulatum* JCM7130 (partial seq. o	527	227	513		3	0	
	M091-09			532	104	237	517	3	3	Y
	M100-01			529	227	515	318	3	0	
	M100-02			529	227	318	515	3	0	
	M109-02			528	513			2	0	
	M148-01			527	512			3	0	
	M148-02			530	237	515		3	2	Y
	M170-02			530	515			3	3	Y
	M170-09			530	238	104	515	3	3	Y
G. vaginalis	M031-02	Y	*Gardnerella vaginalis* ATCC14018 88%	235	459			0	0	
	M045-03	Y	*Gardnerella vaginalis* ATCC14018 89%	459	444			0	0	
	M052-05	Y	*Gardnerella vaginalis* ATCC14018 89%	455	269	440		1	1	
	M067-07	Y	*Gardnerella vaginalis* ATCC14018 89%	453	438			1	2	
	M072-03	Y	*Gardnerella vaginalis* ATCC14018 88%	460	235	445		1	1	
	M077-04	Y	*Gardnerella vaginalis* ATCC14018 90%	457	442	232	109	0	0	

Table S1: Complete list and phylogenetic/phenotypic details of isolates

Group	Isolate	Seq'd	cpn60 UT Nearest Neighbour	ISR peaks				Phenotype		
				Major	Minor1	Minor2	Minor3	Acid+	H_2O_2+	Double-strong
G. vaginalis (cont.)	M082-02	Y	*Gardnerella vaginalis* ATCC14018 88%	457	162	442		1	1	
	M103-06	Y	*Gardnerella vaginalis* ATCC14018 88%	454	439	199	108	0	0	
	M103-07	Y	*Gardnerella vaginalis* ATCC14018 89%	454	334	439		0	0	
	M126-06	Y	*Gardnerella vaginalis* ATCC14018 88%	460	445			0	1	
	M139-06	Y	*Gardnerella vaginalis* ATCC14018 88%	458	235	443		0	0	
	M157-06	Y	*Gardnerella vaginalis* ATCC14018 90%	464	449	235		0	0	
	M163-04	Y	*Gardnerella vaginalis* ATCC14018 99%	232	321	455	313	1	0	
	M165-01	Y	*Gardnerella vaginalis* ATCC14018 97%	455	164	440		0	0	
	M176-02	Y	*Gardnerella vaginalis* ATCC14018 88%	455	440			0	0	
	M176-03	Y	*Gardnerella vaginalis* ATCC14018 90%	456	441	235		0	2	
	M207-05	Y	*Gardnerella vaginalis* ATCC14018 88%	502	458	175	619	3	2	Y
	M220-04	Y	*Gardnerella vaginalis* ATCC14018 89%	453	459	438		0	0	
	P006-02	Y	*Gardnerella vaginalis* ATCC14018 89%	454	439			0	0	
	M022-06			458	443			1	1	
	M029-06			439	454			0	0	
	M031-01			459	235	444	218	0	0	
	M031-03			459	235	444	218	0	0	
	M031-05			459	235	444		1	0	
	M031-06			459	444			0	0	
	M045-02			459	164	444	234	0	0	
	M045-04			459	444			0	0	
	M045-05			460	235	110	445	0	0	
	M059-04			459	234	443		0	0	
	M061-01			455	281	214	440	0	0	
	M072-04			460	235	445		0	1	
	M076-03			457	442			1	0	
	M083-06			455	440	281	426	0	1	
	M101-05			455	440	281		0	0	
	M135-10			456	441			0	0	
	M139-07			454	439			0	0	
	M141-01			459	444	235		0	0	
	M141-02			459	444	235		0	0	
	M141-03			462	456	447		0	0	
	M141-04			462	446			0	0	
	M155-07			460	445	235		0	0	
	M157-05			456	441			0	0	
	M158-03			453	460	438	445	0	0	
	M158-04			453	460	438	445	0	0	
	M161-08			459	444			0	2	
	M163-05			455	440			0	2	

Table S1: Complete list and phylogenetic/phenotypic details of isolates

Group	Isolate	Seq'd	cpn60 UT Nearest Neighbour	ISR peaks				Phenotype		
				Major	*Minor1*	*Minor2*	*Minor3*	*Acid+*	*H_2O_2+*	*Double-strong*
G. vaginalis (cont.)	M163-06			466	451			0	0	
	M163-07			466	451			0	0	
	M166-04			456	441			0	0	
	M170-08			438	453			0	0	
	M175-03			460	445			0	0	
	M176-04			456	441	235		0	2	
	M178-07			457	441			1	2	
	M187-01			458	443			0	0	
	M187-02			456	441			0	0	
	M187-04			456	441			0	0	
	M190-04			455	440			0	0	
	M190-05			460	445			0	0	
	M199-05			454	439			0	0	
	M200-06			458	443			0	0	
	M200-08			454	439			0	0	
	M205-08			454	439			0	0	
	M215-06			459	444			0	0	
	M225-09			453	438			0	0	
	P006-03			454	438			0	0	
	P009-12			457	442			0	1	
	P030-05			457	162	442		1	1	
	P030-06			457	442			1	1	
Unidentified (Group 1)	M149-01		129/317	129	318	562		2	0	
	M149-02		129/317	562	432	318	538	2	0	
	M149-03		129/317	129	317	562	389	2	0	
	M149-04		129/317	129	318	562	389	2	0	
Unidentified (Group 2)	M104-09		210/233	129	210	233	563	2	0	
	P007-01		210/233	210	233	101		2	0	
	P007-02		210/233	210	233			2	0	
	P007-03		210/233	210	233			2	0	
	P007-04		210/233	210	233	562		2	0	
	P007-05		210/233	233	210	101		2	0	
	P007-06		210/233	233	210	562	101	2	0	
	P018-01		210/233	210	318	233	129	3	0	
	P018-02		210/233	210	233			3	0	
	P018-05		210/233	534	210	129	519	3	0	
Unidentified (Group 3)	M012-05		353/245	353	245	338		1	0	
	M012-06		353/245	353	245	338		1	0	
Unidentified (Group 4)	M221-01		523/183	523	183	509	192	3	0	
	M221-04		523/183	523	183			3	0	

Table S1: Complete list and phylogenetic/phenotypic details of isolates

Group	*Isolate*	*Seq'd*	*cpn60 UT Nearest Neighbour*	**ISR peaks**				**Phenotype**		
				Major	*Minor1*	*Minor2*	*Minor3*	*Acid+*	*H_2O_2+*	*Double-strong*
Unidentified (Group 5)	M061-06		526/544	544				1	0	
	M158-02		526/544	541	239	545	526	2	2	
	M175-01		526/544	526				3	0	
Unidentified (Unique)	M057-04		586/333	586	333	349		0	1	
Failed interspacer	M176-01							0	0	
	M225-01							1	1	
	P002-13							0	0	
	P016-06							1	1	
