# Supplementary material for: Selection, Phenotyping and Identification of Acid and Hydrogen Peroxide Producing Bacteria from Vaginal Samples of Canadian and East African Women
Source: PLoS One. 2012 Jul 23;7(7):e41217. doi: 10.1371/journal.pone.0041217 (PMC3402533; doi:10.1371/journal.pone.0041217)
Supplement: Table S2 — Characteristics of individuals providing vaginal samples in Winnipeg (N = 16). (PDF) [file pone.0041217.s002.pdf]

Table S2. Characteristics of individuals providing vaginal samples in Winnipeg (N=16)

| Study# | # samples | BV diagnosis[1] | Sample intervals (days) | Age[2] | Sexually active[2] | Birth control[2] |
|---|---|---|---|---|---|---|
| 1 | 4 | 1,1,1,1 | 7,14,7 | 14 | N | N |
| 2 | 4 | 3,1,1,1 | 7,7,7 | 14 | N | N |
| 5 | 3 | 3,3,3 | 14,7 | 15 | N | N |
| 6 | 1 | 3 | - | 15 | Y | Oral |
| 7 | 1 | 3 | - | 16 | Y | Transdermal |
| 9 | 2 | 1,1 | 8 | 14 | Y | Injection |
| 11 | 2 | 1,2 | 7 | 14 | Y | Injection |
| 13 | 3 | 1,1,1 | 15,21 | 16 | Y | Intravaginal |
| 14 | 3 | 2,1,1 | 9,7 | 17 | Y | Injection |
| 16 | 4 | 2,1,1,2 | 12,8,9 | 17 | Y | Injection |
| 18 | 3 | 3,1,3 | 16,12 | 15 | Y | Oral |
| 21 | 3 | 2,1,2 | 7,7 | 18 | Y | Oral |
| 22 | 1 | 3 | - | 15 | Y | N |
| 25 | 2 | 3,3 | 8 | 17 | Y | N |
| 30 | 3 | 1,3,2 | 8,7 | 14 | Y | Transdermal |
| 31 | 3 | 3,1,1 | 7,7 | 16 | N | N |

[1] BV diagnosis by Gram stain analysis at each sampling: 1 = BV-, 2=BV-intermediate,

[2] Self-reported on written questionnaire
