# Supplementary material for: Selection, Phenotyping and Identification of Acid and Hydrogen Peroxide Producing Bacteria from Vaginal Samples of Canadian and East African Women
Source: PLoS One. 2012 Jul 23;7(7):e41217. doi: 10.1371/journal.pone.0041217 (PMC3402533; doi:10.1371/journal.pone.0041217)
Supplement: Table S3 — Characteristics of individuals providing vaginal samples in Nairobi (N = 96). (PDF) [file pone.0041217.s003.pdf]

Table S3. Characteristics of individuals providing vaginal samples in Nairobi (N=96)

Serostatus	
HIV-	64 (67%)
HIV+	32 (33%)
Age	
Median	34
Range	21-56
Current sex work	
Active	87 (91%)
Stopped	9 (9%)
Menopause	
Yes	3 (3%)
No	92 (96%)
Missing	1 (1%)
Pregnant	
Yes	2 (2%)
No	93 (97%)
Missing	1 (1%)
Hormonal contraception	
No	68 (71%)
Oral	10 (10%)
Injection	16 (17%)
Missing	2 (2%)
Vaginal symptoms[1]	
Any	10 (10%)
Missing	1 (1%)
Other diagnoses[2]	
Any	2 (2%)
Missing	1 (1%)
Current sexually transmitted infections	
Syphilis	5 (5%)
Gonorrhea	2 (2%)
Trichomonas	3 (3%)
Any	10 (11%)
Missing	1 (1%)
BV diagnosis[3]	
BV-	42 (44%)
BV-intermediate	26 (27%)
BV+	28 (29%)

[1] Including discharge, itch and dysuria

[2] Including ulcers, candidiasis, cystitis, cervicitis

[3] BV diagnosis by Gram stain analysis
